# A Novel Test for Absolute Fit of Evolutionary Models Provides a Means to Correctly Identify the Substitution Model and the Model Tree

**DOI:** 10.1093/gbe/evz167

**Published:** 2019-08-01

**Authors:** Vadim Goremykin

**Affiliations:** Research and Innovation Centre, Fondazione Edmund Mach, San Michele all'Adige, Trentino, Italy

**Keywords:** molecular evolution, phylogenetics, model-data fit, evolutionary models

## Abstract

A novel test is described that visualizes the absolute model-data fit of the substitution and tree components of an evolutionary model. The test utilizes statistics based on counts of character state matches and mismatches in alignments of observed and simulated sequences. This comparison is used to assess model-data fit. In simulations conducted to evaluate the performance of the test, the test estimator was able to identify both the correct tree topology and substitution model under conditions where the Goldman–Cox test—which tests the fit of a substitution model to sequence data and is also based on comparing simulated replicates with observed data—showed high error rates. The novel test was found to identify the correct tree topology within a wide range of DNA substitution model misspecifications, indicating the high discriminatory power of the test. Use of this test provides a practical approach for assessing absolute model-data fit when testing phylogenetic hypotheses.

## Introduction

Substitution model misspecification contributes greatly to phylogenetic uncertainty (Gaut and Lewis 1995; Inagaki et al. 2004; Jermiin et al. 2004; Gruenheit et al. 2008; Sheffield et al. 2009; Nesnidal et al. 2010; Betancur-R et al. 2013; Goremykin et al. 2013, 2015; Xi et al. 2014) and can lead to phylogenetic error in reconstructed relationships (Lockhart et al. 1996; Bruno and Halpern 1999; Buckley 2002; Lartillot et al. 2007). However, direct estimations of model-data fit are very rarely used in phylogenetic practice. In the vast majority of exploratory phylogenetic studies, only the relative fit of different substitution models to each other is estimated (e.g., via the hierarchical likelihood ratio tests: Frati et al. 1997; Sullivan et al. 1997; via Akaike or Bayesian information criterion-based comparisons, respectively: Akaike 1974; Schwarz 1978; or via cross-validation: Lartillot et al. 2007). This practice occurs, even though there are strong indications that assessment of model-data fit based on direct comparison of observed data and parametric replicates is more reliable for assessing phylogenetic accuracy than are measures of relative model fit (Waddell et al. 2009; Grievink et al. 2010).

Absolute model fit indicators compare simulated and empirical data, and the measures of resemblance used by different approaches are diverse. A characteristic trait of absolute model fit indicators is that the best possible model has a fit of zero. All indicators assess whether an evolutionary model is likely to well predict observed data properties. A weakness of current methods is that the statistics employed are often insufficient to distinguish between models. The most widely used statistics are the frequentist Goldman–Cox (GC) test (Goldman 1993) and the Bayesian posterior predictive model checking approach (Rubin 1984; Bollback 2002). Both methods utilize multinomial likelihood-based statistics to assess absolute model fit (Althoff et al. 2006; Lanfear and Bromham 2008; Grievink et al. 2010; Anisimova et al. 2011; Ekman and Blaalid 2011; Nguyen et al. 2012; Reid et al. 2014; Kitahara et al. 2014; Kaehler et al. 2015; Duchêne et al. 2016). These two tests assess overall model fit, and not specific data features such as composition and saturation (Foster 2004; Duchêne et al. 2016). These tests are also similar in the sense that they check the ability of a model to correctly predict the distribution of frequencies of site patterns in the observed data.

The statistics of the frequentist GC test examine the difference between the model-based likelihood and the multinomial likelihood (the product, across all alignment positions, of frequencies of site patterns). Although multinomial likelihoods are simple to calculate they have also been reported to lack discriminatory power (Bollback 2002; Foster 2004; Waddell et al. 2009; Ripplinger and Sullivan 2010; Duchêne et al. 2016).

Lewis et al. (2014) have noted that multinomial likelihood-based statistics “depend[s] only on the counts associated with site patterns and not the patterns themselves. Thus, two data sets that are extremely different can potentially have identical numerical values for this statistic.” Pointing out the irrelevance of information contained in the site patterns for the multinomial likelihood inference, Lewis et al. (2014) have instead suggested a modification of the Gelfand and Ghosh (1998) test statistic (*GG*) for assessment of similarity between observed data and replicates simulated on an evolutionary model. An advantage of using this statistic is that it evaluates accuracy in prediction of data patterns directly (Lewis et al. 2014). Theoretically, the *GG* test statistic as applied by Lewis et al. (2014) can evaluate model-based predictions of distinct site pattern frequencies. Practically, the test requires that the same site pattern must occur at least once in the observed data and in each simulated replicate in order to avoid calculation of logarithm of zero (as explained in detail in the section “Estimation of Substitution Model Fit”). Because this is unlikely to always occur, Lewis et al. (2014) group individual patterns into categories composed of *A*, *C*, *G*, *T*, *AC*, *AG*, *AT*, *CG*, *CT*, *GT*, *ACG*, *ACT*, *AGT*, *CGT*, and *ACGT* character states, since these categories are more likely to be encountered in all alignments.

To further explore the potential of using phylogenetic information contained in alignment site patterns, a novel pattern-sensitive frequentist test is proposed for assessing absolute model-data fit. This test incorporates *GG* statistics, albeit based on different data properties. The discriminatory power of this novel test for model-data fit is compared with the frequentist GC test. The performance of these tests has been examined for a number of DNA evolutionary models. Because phylogenetic analyses of biological data always involve a degree of model misfit, assessment of the tests has considered model-data fit when the substitution model component of the evolutionary model was misspecified. The novel test metric described was found to be sufficiently sensitive to identify the correct tree topology within wide range of DNA substitution model misspecification. The high discriminatory power of the novel test and its robustness to substitution model misspecification encourage its use for hypothesis testing in phylogenetics.

## Materials and Methods

### Calculation Details for Testing and Ranking Models

#### Estimation of Substitution Model Fit

In order to perform the test of substitution model fit, counts are first made of pairwise aligned character states (e.g., *A–A*, *A–C*, *A–G*, *A–T*, *C–C*, *C–G*, *C–T*, *G–G*, *G–T*, *T–T*) in each multiple sequence alignment analyzed. For a multiple sequence alignment (henceforth referred as msa) which has *n* sequences and *k* columns the counts of character state alignments are calculated in the following way:

(1) $$C_{\mathrm{xy}}=\sum_{a=1}^{a=k}\sum_{i=1}^{i=n}\sum_{j=1}^{j=n}1_{i\neq j}1_{\mathrm{Sia}=x}1_{\mathrm{Sja}=y},$$

wherein *x*∊{*A, C, G, T*}, *y*∊{*A, C, G, T*}, *S_ia_* and *S_ja_* are the character states at site a for sequences *i* and *j*, respectively, and **1**_*v*_ = 1 if v is true and 0 otherwise. The alignments compared should not contain gaps and ambiguous character states.

The counts of pairwise aligned character states (eq. 1) are quantities which can be expected to be predicted well by a fit substitution model and poorly when a substitution model is misspecified. They represent the basic type of information used by the proposed test. The test statistic is calculated by comparing the counts of pairwise aligned character states in the data, which have evolved under an unknown model of evolution (henceforth referred as the “empirical model,” EM), to the counts in replicates simulated under an explicit evolutionary model (henceforth referred as the “simulation model,” SM).

The *GG* criterion for model fit is a sum of two components, one related to goodness-of-fit (*GGg*) and the other related to variance (*GGp*):

(2) GG=GGg+GGp.

For the purposes of conducting the test, the default *GG* parameters as recommended in Lewis et al. (2014) following Ibrahim et al. (2001) and Chen et al. (2004) are used. If the sum of all counts of pairwise aligned character states in each msa included in the test is *s*, and the number of replicates generated is *q*, then

(3) $$\mathrm{GGp}=2s\left[\left(\frac{1}{q}\sum_{i=1}^{i=q}t_{1\left(i\right)}\right)-t_{2}\right]$$

and

(4) $$\mathrm{GGg}=4s\left(\frac{t_{2}+t_{3}}{2}-t_{4}\right)$$.

Each of the four *t* functions in equations (3) and (4) is computed as:

(5) $$t_{x}=-\text{ln}s+\frac{1}{s}\sum_{i=1}^{i=d}C_{\left(i\right)}\text{ln}C_{\left(i\right)},$$

wherein *x* represents the corresponding function number as shown in equations (3) and (4), *d* is an alphabet-specific number of distinct character state alignments and *C* is a variable indicating the values for counts of character state alignments. For *t*_1_, the *C* variable indicates the values for character state alignment counts in each replicate. For *t*_2_, the *C* variable indicates the mean value over counts of each such alignment in the distribution of replicates. For *t*_3_, the *C* variable indicates the counts of pairwise character state alignment in the observed msa. In the *t*_4_ function, the *C* variable indicates the mean value for each character state alignment over two corresponding values used in the functions *t*_2_ and *t*_3_.

Although originally suggested for Bayesian analyses (Gelfand and Ghosh 1998), these statistics in fact can also be expected to function well in a frequentist analytical framework. In the case of no variation in the counts among simulated replicates, the indicator of variance (*GGp*) would equal zero. In addition, a perfect fit (*GGg* = 0) occurs when the mean values predicted by the SM do not deviate from those of the empirical model. Obviously, such definitions of fit and variance should hold true for comparisons of observed data with parametric replicates generated under maximum likelihood models too.

Since use of the full *GG* statistic (*GG* = *GGg*+*GGp*) offered no sizable advantage over using only the *GGg* function in the experiments conducted here, the proposed test uses *GGg* function as a default option. The full *GG* statistic, although not recommended, can also be optionally employed.

An important advantage of the *GGg*-based test of substitution model fit presented here over the test suggested in Lewis et al. (2014) is that the *GGg*-based test can be performed under conditions where the Lewis et al. test cannot be applied. Regardless of the data properties compared, Gelfand and Ghosh statistics require each *C* variable in equation (5) to have a positive value in order to avoid calculation of logarithm of zero. In the case of Lewis et al. test, the *C* variable indicates counts for 15 site pattern categories which contain the following character states: A, C, G, T, AC, AG, AT, CG, CT, GT, ACG, ACT, AGT, CGT, and ACGT. These categories should be present in observed data and in each replicate, otherwise the test cannot be performed. The test presented here requires the following ten character state alignments (in any combination of character states, since *C_xy_* = *C_yx_* [eq. 1]): A–A, C–C, T–T, G–G, A–C, A–G, A–T, C–G, C–T, G–T to co-occur in the observed data. They must also occur at least once in the distribution of replicates. Thus, the more replicates that are generated, the higher is the chance of failure in calculation of the Lewis et al. test and the lower is the chance of failure in calculation of the proposed test. Moreover, the alignments of character states can be distributed across the above site pattern categories in any order. This leads to fewer categories necessary to conduct the proposed test compared with the Lewis et al. test. For example, a single ACGT category contains all necessary alignments of nonequal character states and can also contain all other alignments. Simpler AC, AG, AT, CG, CT, and GT categories contain all necessary alignments of nonequal character states and are likely to contain all other alignments too. As a consequence, the suggested test can be performed when Lewis et al. test cannot be employed.

In preliminary experiments aimed at working out the scoring options in the presented study, I encountered program crashes which could be traced back to the problem of noncalculability of Lewis et al. test with large number of replicates and small alignments used for testing purposes. For this reason, this test cannot be performed on a number of published data sets (Nikiforova et al. 2013; Fučíková et al. 2016; Johnson et al. 2018; McManus et al. 2018). The alignments from these studies which cause calculation failure of the test (43 in total) are provided as Supplementary Material online. In contrast to the performance of Lewis et al. test, these alignments, ranging in length from 96 to 134,553 positions, fulfill the conditions required by the novel test presented here.

**Fig. 1.**
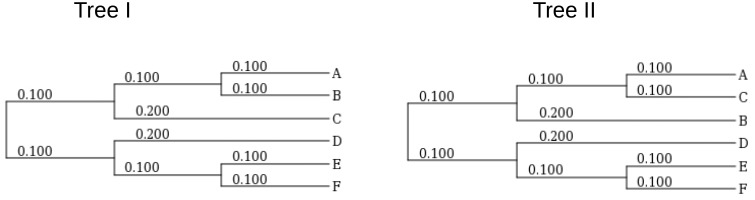
—Model trees used for simulation.

Assessment of substitution model fit based on counts of pairwise alignments among the character states is illustrated with the following example. Let us consider the two trees shown in figure 1. In Tree I, taxon A is sister to taxon B, while in Tree II, taxon A is sister to taxon C, otherwise the trees are identical.

With the help of the INDELIBLE program (Fletcher and Yang 2009), a 10,000 positions long replicate has been simulated on Tree I. The simulation assumed a GTR+G model (with the numerical parameters taken from arbitrarily selected T01+GTR+G EM model used in table 2). The corresponding evolutionary model is referred to hereafter as the “empirical model 1,” EM1. Two further replicates of the same length were generated under Tree I and Tree II assuming a GTR model with the same numerical parameters as the above GTR+G model. These evolutionary models assuming Tree I and Tree II have been termed simulation model 1 (SM1) and simulation model 2 (SM2), respectively. SM1 was used to illustrate a case where the tree and substitution model are correct, while SM2 illustrates a case where the tree is misspecified and the substitution model is correct. Replicates of the same length were also generated under Tree I and Tree II assuming a K80 model (with the numerical parameters taken from arbitrarily selected T01+K80 EM model used in table 2). These evolutionary models assuming Tree I and Tree II have been termed simulation model 3 (SM3) and simulation model 4 (SM4), respectively. SM3 was used to illustrate a case where the tree is correct and the substitution model is misspecified. SM4 illustrates a case where both the tree and substitution model were misspecified.

**Table 2:** **Ability of the Presented Test to Identify the Tree Components of the Preferred Evolutionary Models** ^a^ **in the Presence and Absence of Model Misspecification**

(A) Estimates of Fit^b^ of the Preferred Models to GTR+R-Based Empirical Models										
EMs	GTR+R	GTR+G	HKY+G	GTR+G+I	K80+G	HKY+G+I	K80+G+I	K80	HKY	GTR
T01+GTR+R	72	140,104	140,577	139,809	135,923	140,867	134,071	136,622	148,437	161,611
T02+GTR+R	69	131,197	131,783	131,565	130,572	131,403	128,777	132,168	141,760	154,265
T03+GTR+R	71	130,215	130,868	130,249	130,569	130,551	128,288	130,691	139,927	151,887
T04+GTR+R	68	143,709	143,204	144,926	138,741	145,687	137,618	138,434	151,773	166,060
T05+GTR+R	67	137,612	138,250	136,837	133,771	137,028	132,294	133,530	145,973	158,175
T06+GTR+R	43	107,902	111,203	110,976	119,041	113,739	119,891	123,072	128,937	138,872
T07+GTR+R	43	104,752	107,363	107,085	114,292	108,468	115,759	117,707	124,410	134,180
T08+GTR+R	42	102,443	103,521	105,366	111,828	106,335	113,743	115,196	121,314	131,279
T09+GTR+R	47	110,833	111,923	113,969	117,759	114,943	120,343	122,367	131,718	142,798
T10+GTR+R	46	105,652	107,640	108,651	114,748	110,930	116,885	118,177	125,811	137,070
T11+GTR+R	48	108,982	110,953	112,019	113,490	114,422	115,248	119,651	135,052	144,390
T12+GTR+R	44	103,037	104,921	105,148	107,660	108,741	108,968	113,336	125,545	135,570
T13+GTR+R	43	102,781	102,768	104,088	106,104	106,491	107,766	110,640	122,711	132,414
T14+GTR+R	52	112,073	112,888	114,884	113,350	118,273	118,473	120,560	137,952	147,677
T15+GTR+R	48	105,540	107,818	108,270	108,549	109,886	111,381	114,085	130,567	140,314


(B) MS Values^c^ for the Preferred Models Shown in Subtable A										
EMs	GTR+R	GTR+G	HKY+G	GTR+G+I	K80+G	HKY+G+I	K80+G+I	K80	HKY	GTR
T01+GTR+R	94	93	94	96	96	95	96	93	90	91
T02+GTR+R	98	98	98	98	98	98	98	98	98	98
T03+GTR+R	99	99	99	99	98	99	98	98	99	99
T04+GTR+R	86	86	86	83	82	83	79	86	87	88
T05+GTR+R	99	99	99	99	99	99	99	99	99	99
T06+GTR+R	95	94	93	95	92	93	95	90	93	93
T07+GTR+R	98	97	96	98	96	97	96	96	97	97
T08+GTR+R	99	98	99	98	98	98	98	97	99	98
T09+GTR+R	86	88	91	87	92	90	89	94	91	89
T10+GTR+R	99	100	100	100	99	100	99	100	100	100
T11+GTR+R	96	95	94	95	93	96	96	92	94	93
T12+GTR+R	97	98	98	98	98	97	98	98	98	98
T13+GTR+R	98	97	97	97	96	98	96	96	97	97
T14+GTR+R	85	86	86	86	88	82	83	90	87	89
T15+GTR+R	100	100	100	100	99	100	100	99	100	100


(C) Estimates of Fit^b^ of the Preferred Models to GTR+G-Based Empirical Models										
EMs	GTR+G	GTR+G+I	HKY+G	HKY+G+I	HKY	GTR	K80+G	K80+G+I	K80	GTR+R
T01+GTR+G	51	74	2,043	2,105	15,462	16,739	23,677	23,562	27,833	140,851
T02+GTR+G	47	69	1,975	2,044	15,499	16,718	23,590	23,588	28,380	133,976
T03+GTR+G	49	71	1,961	2,019	15,162	16,367	23,196	23,101	27,478	132,028
T04+GTR+G	53	76	2,112	2,217	16,236	17,995	24,764	24,817	29,260	143,301
T05+GTR+G	48	72	2,067	2,136	15,822	17,177	24,256	24,211	28,611	137,211
T06+GTR+G	37	53	1,575	1,665	14,247	15,437	20,246	20,819	25,754	107,128
T07+GTR+G	34	51	1,530	1,617	14,050	15,065	19,553	20,101	24,690	104,006
T08+GTR+G	35	51	1,482	1,588	13,631	14,849	18,913	19,772	23,998	102,077
T09+GTR+G	40	56	1,581	1,675	14,418	15,787	20,348	21,133	25,694	107,947
T10+GTR+G	36	53	1,509	1,631	13,917	15,136	19,465	20,147	24,451	103,195
T11+GTR+G	36	53	1,494	1,590	14,194	15,297	18,259	18,879	23,572	108,964
T12+GTR+G	34	49	1,425	1,526	13,316	14,571	17,906	18,439	22,876	101,202
T13+GTR+G	33	49	1,415	1,507	13,422	14,553	17,504	18,106	22,571	100,703
T14+GTR+G	38	55	1,582	1,710	14,866	15,990	19,329	20,491	24,829	112,148
T15+GTR+G	36	53	1,505	1,582	14,051	15,201	18,166	18,985	23,405	105,331


(D) MS Values^c^ for the Preferred Models Shown in Subtable C										
EMs	GTR+G	GTR+G+I	HKY+G	HKY+G+I	HKY	GTR	K80+G	K80+G+I	K80	GTR+R
T01+GTR+G	98	99	98	98	96	97	99	99	98	97
T02+GTR+G	99	100	99	99	99	99	100	100	99	99
T03+GTR+G	99	99	100	100	99	99	99	99	99	100
T04+GTR+G	91	89	89	89	93	91	86	84	90	91
T05+GTR+G	100	100	100	100	100	100	100	100	100	100
T06+GTR+G	97	97	96	97	96	96	97	97	95	97
T07+GTR+G	98	98	98	98	97	99	98	98	98	97
T08+GTR+G	99	99	99	99	99	99	99	99	99	99
T09+GTR+G	93	94	94	94	94	93	92	91	95	93
T10+GTR+G	100	100	100	100	100	100	100	100	100	100
T11+GTR+G	97	97	96	98	96	95	97	98	95	97
T12+GTR+G	99	99	98	98	98	98	99	99	99	98
T13+GTR+G	99	98	99	99	99	99	98	98	98	99
T14+GTR+G	90	89	90	89	92	94	90	85	94	90
T15+GTR+G	100	100	100	100	100	100	100	100	100	100


a Preferred simulation models assume the correct tree topology of the empirical model (EM, shown in leftmost column) and one of the ten substitution model components shown above the subtables. Tree topologies of 15 full topological constraints being part of the EMs are presented in supplementary figure S1, Supplementary Material online.

b The mean model fit estimates between each of the 500 replicates representing an empirical model (EM) and a preferred SM.

c The percentages of times when a preferred SM showed better fit to the each of 500 replicates representing EM in comparison to a distinct SM assuming the same substitution model scheme as the preferred model. Shown are the worst (lowest) MS values for the separation of a preferred SM from any other SM which assumes the same substitution model scheme.

**Fig. 2.**
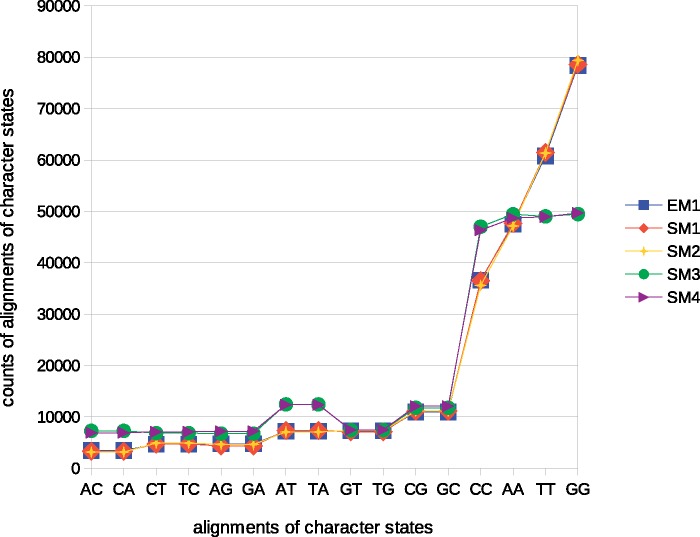
—Changes in the distributions of the counts of character state alignments resulting from substitution model misspecification. In the figure, EM1 and SM1 assume Tree I (fig. 1) plus GTR+G model, SM2 assumes Tree II (fig. 1) plus GTR+G model, SM3 assumes Tree I plus K80 model and SM4 assumes Tree II plus K80 model.

Counts of alignments among character states (eq. 1) in replicates representing EM1, and four SMs were calculated (fig. 2). The results presented in figure 2 show similarity in the counts calculated for the replicates simulated under all models assuming a GTR+G substitution model component (EM1, SM1, and SM2). The values calculated for the models assuming a K80 substitution model component (SM3 and SM4) were also similar to each other. These were quite distinct from those calculated for GTR+G-based replicates for a majority of character state alignments. *GGg* test values calculated in comparisons of EM1 to SM1 (68.14), EM1 to SM2 (69.76), EM1 to SM3 (16,367.30), and EM1 to SM4 (16,135.76) allow visualization of the difference in data-model fit due to substitution model misspecification.

The test component used for assessing overall substitution model fit is a mean over *GGg* values computed for a set of evolutionary models sharing a substitution model component:

(6) $$A=\frac{1}{w}\sum_{i=1}^{i=w}\mathrm{GG}g_{i},$$

wherein *w* is a number of such models. The tree model component influences *GGg* values only slightly and randomly, and so this metric cannot be used to rank alternative tree components of an evolutionary model. For example, SM3 which shared the same tree with EM showed worse fit compared with SM4, an evolutionary model which assumed a wrong tree. In the example presented above, the *A* value (eq. 6) for a GTR+G model is calculated as (68.14 + 69.76)/2 = 68.95 and the analogous value for a K80 model is calculated as (16,367.30 + 16,135.76)/2 = 16,251.53.

#### Ranking Combinations of Each Substitution Model and Model Tree in Terms of Model-Data Fit

The metric presented above cannot be used to rank alternative tree components of an evolutionary model. However, by considering data partitions (groups of sequences) separately, a further statistic can be defined that will provide a means of accomplishing this task.

To obtain this statistic, the counts of pairwise aligned character states are first transformed into frequencies. For each msa which has *n* sequences and *k* columns:

(7) $$f_{\mathrm{xy}}=\frac{C_{\mathrm{xy}}}{\mathrm{kn}\left(n-1\right)},$$

wherein *x*∊{A, C, G, T}, *y*∊{A, C, G, T}, and *C_xy_* is the value estimated in equation (1). The frequencies of alignments of nonequal character states should be divided by a large constant positive value (P-factor) to produce the scoring matrix values. As explained later in this section, division of frequencies of character state mismatches by a large positive P-factor value improves the discriminatory power of the test. The scoring matrix values are given by equation (8):

(8) $$m_{\mathrm{xy}}=\frac{f_{\mathrm{xy}}}{P_{x\neq y}},$$

wherein **P**_*v*_ = *P* if *v* is true and 1 otherwise. The script which performs the calculation for the component of the presented test related to model tree fit (test_stage1.pl, available at: https://github.com/vadimgoremykin/absolute-model-data-fit) uses a default P-factor value = 10,000, which was used in all experiments conducted here, unless stated otherwise.

The scoring matrix values are used to estimate dissimilarity (*D* values) between subsets of taxa separated by internal branches in the model trees. *D* values provide a means to evaluate which fully resolved tree is most supported by the data. Furthermore, although the space of all possible trees can be large, competing hypotheses of relationship often only concern a finite number of possible tree topologies, which are represented by a limited number of alternative internal branches. Thus, while there are 2^(^^*n*^^−^^1)^ −*n* −1 splits or internal branches in a tree space with *n* taxa, *D* values do not need to be calculated for all splits in the possible tree space, but rather they need only be calculated for a set of splits that represents competing trees. *D* values can be used to rank tree models because their value is influenced by the treelike divergence of sequences.

*D* values for each internal branch are obtained by considering the average heterogeneity of *m* values for the node on one side of the branch being evaluated against the average heterogeneity of *m* values across the branch at each alignment position. A *D* value is calculated for each of the two internal tree nodes separated by the internal branch. The position-based *D* value for each node is calculated as the ratio of a mean m value (eq. 8) for character alignments within the taxon subset on a side of the branch corresponding to the node (termed here “node-specific taxon subset”) at the given alignment column c (*V*1_c_) to the mean m value for alignments between these characters and all other characters at the same column (*V*2_c_).

The *V*1_c_ value is computed as the mean of the *m* values (eq. 8) for all possible alignments between the character states in the node-specific taxon subset at the column c. Since *m_xy_* = *m_yx_*, *V*1_c_ can be computed as a mean over the *m* values in an upper or lower triangular part of a square matrix which rows and columns represent OTUs included into the subset, populated with the m values for letter alignments among these OTUs, e.g.:

(9) $$\begin{array}{c} V1_{c}=\frac{1}{h\left(h-1\right)0.5}\sum_{i=1}^{i=h}\sum_{j=1}^{j=h}m_{x(i),y(j)}\\ \;\;\;\;\;\;\;\;\;\;\;\;\;\;\;\;\;(i\;>\;j) \end{array},$$

wherein *h* is the number of taxa in the subset, and *m_x_*_(__*i*__),__*y*__(__*j*__)_ is the scoring matrix value for the character states in column c (*x* and *y*) observed, respectively, in sequences *i* and *j* which belong to the subset.

The value for *V*2_c_ is analogously computed as a mean of *m* values for the character state alignments between each character state observed in the node-specific taxon subset at the same column c and each character state not included in the subset at the column:

(10) $$V2_{c}=\frac{1}{\mathrm{hb}}\sum_{i=1}^{i=h}\sum_{j=1}^{j=b}m_{x(i),y(j)},$$

where *x*(*i*) is a character in the column c observed at the *i*th OTU from the node-specific taxon subset which has a total of *h* OTUs, *y*(*j*) is a character in the column c observed at the *j*th OTU which belongs to the rest of OTUs, comprised of *b* sequences and *m_x_*_(__*i*__),__*y*__(__*j*__)_ is the scoring matrix value for *x*(*i*) and *y*(*j*) character states.

The *D* value for a node-specific taxon subset at the column c is a ratio of the corresponding *V*1_c_ value to the *V*2_c_ value:

(11) $$D_{c}=\frac{V1_{c}}{V2_{c}}.$$

The *D* value for a node-specific taxon subset in a multiple sequence alignment of *k* positions is calculated as the arithmetical mean of all the corresponding position-based *D_c_* values:

(12) $$D=\frac{1}{k}\sum_{c=1}^{c=k}D_{c}.$$

The *D* values are computed for all internal tree branches present in all the model trees representing alternative evolutionary hypotheses to be tested. For the test purposes, the trees should be fully resolved. The set of the *D* values (eq. 12) used for model fit assessment should not contain redundant estimates. Regardless of the frequency of occurrence of an internal branch in the model trees, the corresponding *D* values should be present in the set only once.

A feature of this novel statistics which is utilized to identify the true tree topology is that *D* values that correspond to splits that are in the true tree will tend to be larger than *D* values for the same splits if they are not in the true tree. Employing P-factors in calculation of *D* values helps to emphasize this difference.

In designing a scoring function related to model tree fit, it was assumed that common character state(s) observed across a hypothesized branch at an alignment position indicate that the position (termed “X site”) does not support the branch, but rather supports other branches or does not support any branch (e.g., is constant). In addition, it is assumed that a position at which common character state(s) are not shared across a hypothesized branch (termed “S site”) supports the branch. In the case of S sites, the denominator in equation (11) is a mean over the m values assigned to mismatches *only*. The significance of this is that division of frequencies of mismatches by a P-factor value in equation (8) leads to relative increase of sizes of position-based *D* values for S sites compared with the corresponding values calculated for X sites. Consequently, the numerical size of a *D* value (eq. 12), which is a mean of the position-based *D* values calculated over all msa positions, would be largely determined by S sites. Under each substitution model, employing P-factors emphasizes the difference between a *D* value calculated in the case when S sites are abundant, which is expected if a branch is in the true tree, and the *D* value for the *same* node-specific taxon subset calculated when S sites are scarce. The latter is expected when the branch is not in the true tree. The difference between *D* values for the same node-specific taxon subsets expected under these alternative evolutionary scenarios provides the basis for discriminating between model trees as described below. The effect of using P-factors is illustrated in table 3. At high P-factor values, there is greater resolution among models (table 3) while numerical test values (table 5) are similar.

**Table 3:** **An Example of Ability of the Presented Test to Identify Preferred Models** ^a^ **in the Presence and Absence of Model Misspecification as a Function of P-Factor Value Size**

*P* Factor	GTR+R	GTR+G	HKY+G	GTR+G+I	K80+G	HKY+G+I	K80+G+I	K80	HKY	GTR
*P* = 1	87	0	0	0	0	0	0	0	0	0
*P* = 10	71	34	16	13	39	6	1	2	0	0
*P* = 100	79	74	71	80	75	81	83	72	70	73
*P* = 1,000	94	94	94	95	95	94	96	92	88	90
*P* = 10,000	94	93	94	96	96	95	96	93	90	91
*P* = 100,000	94	93	94	96	96	95	96	93	90	91

a Preferred simulation models in this comparisons assume the correct tree topology of the “empirical” model (GTR+R constrained with the full topological constraint congruent to Tree 1 in supplementary fig. S1, Supplementary Material online) and substitution model components shown above the table. The values shown are the percentages of times when a preferred SM showed better fit to the each of 500 replicates representing “empirical” model in comparison to a distinct SM assuming the same substitution model scheme as the preferred model. Shown are the worst (lowest) MS values for the separation of a preferred SM from any other SM which assumes the same substitution model scheme.

**Table 5:** The Test Values Obtained in Comparison of the Empirical Data Set to Full Evolutionary Models Assuming GTR-Based Model with across Sites Rate Heterogeneity Modeled via FreeRate Model (Soubrier et al. 2012) as a Substitution Model Component

	*P* = 1,000	*P* = 10,000	*P* = 100,000
Cladogram 3	247.145	247.164	247.314
Cladogram 4	268.513	268.360	268.512
Cladogram 1	288.913	289.138	289.279
Cladogram 2	305.501	305.355	305.566
Cladogram 5	791.953	791.952	792.194

Note.—Each test value shown in the table was obtained for the evolutionary model assuming model tree topology shown in the leftmost column employing P-factor value (eq. 8) shown above the corresponding column of values. Tree topologies of five full topological constraints used in these experiments (as shown in the leftmost column) are presented in supplementary figure S2, Supplementary Material online.

The *D* values are calculated based on each individual msa, representing either EM or SM. The strength of rank correlation between a set of *D* values calculated based on an alignment representing the empirical model and a set of *D* values calculated for a SM-based replicate is measured using the Spearman correlation coefficient (*r*_s_). The replicate-specific *r*_s_ values are each estimated by comparing a set of *D* values for an individual msa representing EM to a set of *D* values for each replicate representing SM. They are converted to a normally distributed variable *z* by the Fisher transformation:

(13) $$z=\frac{1}{2}\text{ln}\left(\frac{1+r_{s}}{1-r_{s}}\right).$$

In the rare case in which *r*_s_ is >0.999999999999999, *r*_s_ is set to this value to enable computation of the Fisher transformation in the Perl programming language. The arithmetical mean for a q-member distribution of *z* values is calculated as:

(14) $$\bar{z}=\frac{1}{q}\sum_{i=1}^{i=q}z_{i}.$$

The test component used for assessing evolutionary model fit to a msa representing EM is obtained by inverting the Fisher transformation for the mean value (eq. 14) and subtracting the resulting value from 1:

(15) $$B=1-\left(\frac{\;\text{exp}\;\left(2\bar{z}\right)-1}{\;\text{exp}\;\left(2\bar{z}\right)+1}\right).$$

The smaller is the resulting value, the better is SM-data fit.

**Fig. 3.**
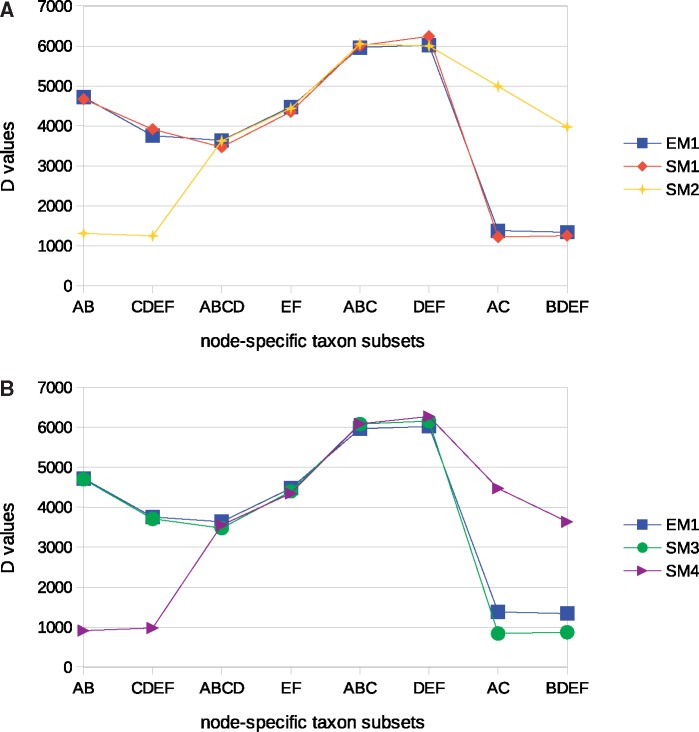
—Evolutionary model-driven changes in the distributions of *D* values calculated for the node-specific taxon subsets corresponding to all the distinct internal tree branches sampled from the model trees (fig. 1). (*A*) Changes registered in absence of substitution model misspecification. In the figure, EM1 and SM1 assume Tree I (fig. 1) plus GTR+G model and SM2 assumes Tree II (fig. 1) plus GTR+G model, (*B*) Changes registered when simulation models shared a misspecified substitution model component. In the figure, SM3 assumes Tree I plus K80 model and SM4 assumes Tree II plus K80 model. Empirical model (EM1) is the same as in figure 3*A*.

Model tree selection based on *D* values is illustrated employing the replicates simulated on the trees shown in figure 1 and used to provide an example for the calculation of substitution model fit (eq. 6). *D* values were calculated based on each replicate for all the node-specific taxon subsets which could be sampled from the model trees (fig. 3).

The results presented in figure 3A and *B* show that, *under each distinct substitution model*, each *D* value that corresponds to a split that is in the model tree is larger than the *D* value for the same node-specific taxon subset if the split is not in the model tree. *D* values for each of the AB and CDEF node-specific taxon subsets which are separated by a branch in the Tree I but not in Tree II are larger for the evolutionary models assuming Tree I compared with those assuming Tree II. *D* values for each of the AC and BDEF node-specific taxon subsets, which are separated by a branch in Tree II but not in Tree I, are smaller for evolutionary models assuming Tree I compared with those assuming Tree II.

Spearman rank correlation coefficients (*r*_s_) were employed to assess the difference in model-data fit due to an incorrect tree. In the case of no substitution model misfit, comparison of identical evolutionary models (EM1 and SM1) yielded *r*_s_ = 0.98, and the comparison of models assuming Tree I and Tree II (EM1 and SM2, respectively) yielded *r*_s_ = 0.38. In comparisons involving GTR+G-based EM1 and evolutionary models that contained a substitution model component (K80 model) that was misspecified, comparison of models assuming the same set of correct branches (EM1 and SM3) yielded *r*_s_ = 0.98, and comparison of EM1 and SM4, assuming different sets, yielded a relatively lower value (*r*_s_ = 0.36).

These results illustrate the assessment of model-data fit when distinct SMs *share the substitution model component* but assume different trees. The highest degree of rank correlation can be expected when tree topologies are equivalent for the empirical model and the simulation evolutionary model. In this case, the sets of correct and wrong branches used for model fit assessment would be the same for the EM and SM. When the SM assumes a misspecified tree, each node-specific *D* value for the internal branch that is in the SM tree, but not in EM tree, is expected to be larger in the simulation than in the observed data. In contrast, each node-specific *D* value for the internal branch that is in the EM tree, but not in the SM tree can be expected to be smaller in the simulation than in the observed data. The rank correlation coefficients provide a simple means to assess these shifts in *D* values in SM compared with EM.

#### Test Statistic

It should be mentioned that the proposed rank correlation analysis is not well-suited to distinguish substitution models which exhibit the same or similar rank correlation values and the same tree topologies. A combined test metric for evolutionary model fit (*T*) which is a product of two components (value *A* and value *B*, eqs. 6 and 15, respectively) is free from this limitation:

(16) T=AB.

In the case of perfect fit the test value should approximate zero. In practice, a fit of 0 is rarely achieved even in comparison of identical models due to stochastic differences in replicate samples.

In the case of comparison of models represented by single replicates used here to illustrate the principles of model fit assessment, *B* = 1−*r*_s_. Thus, comparison of EM1 to identical SM1 yields *T* = 68.95(1–0.98) = 1.38, comparison of EM1 to SM2 yields *T* = 68.95(1–0.38) = 42.75, comparison of EM1 to SM3 yields *T* = 16,251.53(1–0.98) = 325.03 and comparison of EM1 to SM4 yields *T* = 16,251.53(1–0.36) = 10,400.98. The combined test statistic (*T*) was able to identify both the correct substitution model and the model tree. The correct substitution model was identified as a component of the best fitting SM (SM1). The correct model tree could be identified even when the substitution model was misspecified.

### Evaluation and Comparison of Performance

#### Assessment of Resolution among Individual Models and Sufficiency of the Number of Replicates for Correct Model Identification

The discriminatory power of the proposed test has been assessed in experiments involving comparison of explicit models, where correct test outcomes were known. In such experiments, stochasticity in test estimates can arise due to the limited size of the SM-based replicate distribution (error source 1) and due to the choice of the replicate representing the “correct model” (which, e.g., can be an outlier, error source 2). In order to quantify the error rate associated with each specific experimental setup directly, a number of EM-based replicates were compared with each distribution of the SM-derived replicates. When both SM and EM have represented the same evolutionary model, the replicates compared were different.

Iterative comparison of EM-based replicates to a distribution of SM-based replicates can yield the mean test value over all the comparisons, which would be less dependent on the error source 2. Series of such comparisons can also show how well the test can discern one model from another. The parametric bootstrap-based estimate for model separation (MS) employed in this study is the percentage of times a preferred simulation model showed better fit to each EM-derived replicate in comparison to a wrong simulation model.

In the experiments aimed at estimation of the test’s ability to separate each EM from every other model (table 1), the preferred evolutionary models had both components (tree and substitution model) identical to those of the EM. An obvious assumption of these experiments was that the test statistic used should be able to identify the correct model.

**Table 1:** **Summary of Attempts to Identify Each Correct Simulation Evolutionary Model** ^a^ **in a Set of 150 Test Models**

	T1	T2	T3	T4	T5	T6	T7	T8	T9	T10	T11	T12	T13	T14	T15
GTR+G	92.6	89.2	88.8	89.0	90.2	86.6	91.4	89.0	89.6	86.8	90.0	88.0	89.0	88.8	91.0
GTR+R	94.0	97.6	98.8	86.0	99.2	95.4	98.0	98.8	86.4	99.4	95.6	97.4	97.8	85.0	99.6

Note.—The values shown represent the percentage of times when a correct simulation evolutionary model showed better fit to each of 500 replicates representing EM in comparison to any other SM included in analyses. Shown are the worst model separation values registered in analyses.

a Correct simulation evolutionary models assume the correct tree topology (shown above the table and presented in in supplementary fig. S1, Supplementary Material online) and substitution model component of each “empirical” model (shown in leftmost column).

In the experiments aimed at identifying the correct tree topology when the SM substitution model was misspecified, the preferred models had tree topologies that were congruent with the correct tree model. In contrast, unfavored models had incorrect tree topologies under the same substitution models scheme (e.g., K80+G) as in the preferred models. The purpose of these experiments was to test the hypothesis that the specification of a correct tree topology will improve model-data fit in the case when the substitution model is misspecified. In addition, the mean test values for over 500 EM-SM comparisons were also used to assess if the specification of the correct tree topology led to the best estimates of model fit within the SM sets, each characterized by the same misspecified substitution model. Since a degree of model misfit is unavoidable in phylogenetic analyses of the biological data, estimations of the test’s performance under increasingly severe model misspecification (table 2) provides another analytical perspective on the discriminatory power of the test metric.

It should be noted that the MS scores can be calculated for any test of model fit which is based on comparison of replicates. For example, in this study, the MS scores described in the above paragraph were also computed for the GC tests (table 4).

**Table 4:** **Ability of the Goldman–Cox Test to Identify the Tree Components of the Preferred Evolutionary Models** ^a^ **in the Presence and Absence of Model Misspecification**

(A) Estimates of Fit^b^ of the Preferred Models to GTR+G-Based Empirical Models									
Ems	GTR+G	HKY+G	K80+G+I	GTR+G+I	K80+G	HKY+G+I	K80	HKY	GTR
T01+GTR+G	0.80	2.95	3.28	3.64	3.56	8.30	14.17	43.14	45.29
T02+GTR+G	0.83	2.88	3.45	3.91	3.69	7.95	14.14	42.96	43.78
T03+GTR+G	0.78	3.11	3.34	3.54	3.70	8.10	14.42	42.67	43.34
T04+GTR+G	0.82	2.90	3.35	3.38	3.41	8.86	14.70	45.24	47.55
T05+GTR+G	0.82	2.90	3.31	3.51	3.49	7.83	14.73	42.91	44.35
T06+GTR+G	0.85	2.94	2.80	3.11	3.24	7.52	17.61	49.17	45.21
T07+GTR+G	0.73	3.07	2.98	3.18	3.47	8.06	16.23	47.51	46.38
T08+GTR+G	0.80	3.00	2.89	3.02	3.50	7.76	16.16	45.88	49.48
T09+GTR+G	0.80	3.12	3.06	3.04	3.55	7.70	17.31	47.72	48.36
T10+GTR+G	0.84	2.75	2.80	3.17	3.49	7.53	17.14	45.86	48.35
T11+GTR+G	0.82	2.94	3.12	2.86	3.50	7.36	17.41	47.62	46.46
T12+GTR+G	0.76	2.96	3.24	3.25	3.42	7.31	16.59	46.16	47.56
T13+GTR+G	0.85	2.88	3.03	3.18	3.64	7.65	16.75	46.28	46.79
T14+GTR+G	0.73	2.91	2.93	3.23	3.59	7.46	17.54	46.29	48.62
T15+GTR+G	0.75	2.75	2.94	3.15	3.52	7.29	17.30	45.88	46.39

**(B) MS Values^c^ for the Preferred Models Shown in Subtable A**									

**EMs**	**GTR+G**	**HKY+G**	**K80+G+I**	**GTR+G+I**	**K80+G**	**HKY+G+I**	**K80**	**HKY**	**GTR**

T01+GTR+G	53	0	0	0	0	0	0	0	0
T02+GTR+G	12	100	0	0	0	2	100	0	0
T03+GTR+G	51	0	100	66	0	0	0	100	100
T04+GTR+G	46	0	0	29	0	0	0	0	0
T05+GTR+G	39	0	0	0	0	96	0	0	0
T06+GTR+G	3	0	0	0	100	0	0	0	0
T07+GTR+G	7	0	0	0	0	0	0	0	0
T08+GTR+G	42	0	0	0	0	0	0	0	0
T09+GTR+G	35	0	0	0	0	0	0	0	0
T10+GTR+G	29	0	0	0	0	0	0	0	0
T11+GTR+G	22	1	0	1	0	0	0	0	0
T12+GTR+G	50	1	0	0	0	0	0	0	0
T13+GTR+G	1	0	0	0	0	0	0	0	0
T14+GTR+G	51	0	0	0	0	0	0	0	0
T15+GTR+G	54	1	0	0	0	0	0	0	0

a Preferred simulation models assume the correct tree topology of the empirical model (EM, shown in leftmost column) and one of the nine substitution model components shown above the subtables. Tree topologies of 15 full topological constraints being part of the EMs are presented in supplementary figure S1, Supplementary Material online.

b The mean model fit estimates between each of the 500 replicates representing an empirical model (EM) and a preferred SM.

c The percentages of times when a preferred SM showed better fit to the each of 500 replicates representing EM in comparison to a distinct SM assuming the same substitution model scheme as the preferred model. Shown are the worst (lowest) MS values for the separation of a preferred SM from any other SM which assumes the same substitution model scheme.

Increasing the number of replicates (both EM- and SM-based) in experiments aimed at calculation of MS scores can also give a researcher the means to judge whether the degree of model discrimination observed is due to the resolution limit of the method or rather due to stochasticity in replicate sampling (error source 1). Leveling of the MS values obtained in experiments involving incrementally increased replicate numbers would indicate that the former explanation is correct.

#### Assessment of Discriminatory Power for the Novel Test and GC Test

Estimators for model-data fit which use different site pattern-based statistics were evaluated to determine whether they could identify evolutionary models used to simulate parametric replicates. Performance of the estimators was evaluated in instances where there was specification of the correct substitution model and also when there was substitution model misspecification.

Model trees with branch lengths and substitution model parameters for generation of replicates under arbitrarily selected GTR+G_4_, GTR+I+G_4_, GTR, HKY+I+G_4_, HKY+G_4_, HKY, K80+I+G_4_, K80+G_4_, and K80 substitution models were obtained from RaxML v. 825 (Stamatakis 2014) tree searches. For each substitution model, 15 searches under distinct full topological constraints (supplementary fig. S1, Supplementary Material online) were performed based on the 21,300 pos. long alignment of 35 OTUs comprising 30 concatenated aligned mitochondrial gene sequences from land plants and green algae. The alignment was used only to obtain model trees with branch lengths and model numerical parameters.

The trees used as full topological constraints in all the searches performed based on the above-mentioned biological data set were arbitrarily selected to represent various hypotheses of the relationships among the land plants and their closest modern algal relatives. The selection of the constraints simulates a typical case in the phylogenetic literature in which the alternative hypotheses concern changes resulting from pruning and regrafting of several internal branches in competing trees (from one to four, in comparison to every model tree in the experiments presented here).

The best-fitting model for the above data set selected by the ModelFinder pipeline (Kalyaanamoorthy et al. 2017) was a GTR-based model (GTR+R) with across sites rate heterogeneity modeled via FreeRate model (Soubrier et al. 2012), which allows site rates to vary freely and allows automatic determination of the number of rate categories. The best model assumed five rate categories. Model trees and substitution model parameters for generation of replicates under the optimal GTR+R model were obtained from 15 IQ-TREE (Nguyen et al. 2015) searches performed based on the observed alignment iteratively specifying each of the above mentioned 15 topological constraints.

Multiple sequence alignments were simulated using INDELIBLE (Fletcher and Yang 2009) for all the models, except those that used a GTR+R substitution model component, for which I used Seq-Gen (Rambaut and Grassly 1997). All INDELIBLE configuration files used for replicate generation and Seq-Gen command lines used to generate the GTR+R model-based replicate distributions are available as Supplementary Material online.

The simulations under each of GTR+I+G_4_, GTR, HKY+I+G_4_, HKY+G_4_, HKY, K80+I+G_4_, K80+G_4_, and K80-based evolutionary models (substitution model+tree combinations, 120 in total) were conducted to sample sets of 500 parametric replicates, each 21,300 pos. long. The replicate sets were chosen to represent 120 simulation evolutionary models (SMs) in follow up experiments. For each GTR+R and GTR+G_4_-based evolutionary model (30 in total), I simulated 1,000 replicates, each 21,300 pos. long. Each of the 30 resulting replicate files was then partitioned to obtain a partition *A* with the first 500 replicates sampled and a partition *B* containing the rest of replicates. The *A* partitions were chosen to represent 30 “empirical” models (EMs) and the *B* partitions were chosen to represent other 30 simulation evolutionary models (SMs) in follow up experiments.

A number of model trees for EMs differed by rearrangement of very short, adjacent internal branches (as illustrated in supplementary fig. S1, Supplementary Material online, for GTR+G-based EMs). Thus, they provide a good example to test the discriminatory power of the novel test for model-data fit. The novel test was performed to compare each EM replicate to each SM-based replicate distribution. The *T* values (eq. 16) obtained in comparisons of each SM to each individual EM-based replicate were also used to calculate the following: 1) the mean *T* values over 500 comparisons between each EM and each SM and 2) the MS values as the percentage of times when a SM assuming correct tree and substitution model components showed better fit to each EM-based replicate in comparison to any other SM included in analyzes (the results of these experiments are summarized in table 1). The MS values were also used to assess if the specification of the correct tree topology improves model-data fit for the SMs having misspecified substitution model components (the results obtained employing the default P-factor value of 10,000 are presented in the table 2). Analogous experiments were conducted employing P-factor values set to 1, 10, 100, 1,000, and 100,000 for a subset of evolutionary models to illustrate the effect of P-factor values on ability of the test to identify correct tree topology (table 3).

The discriminatory power of the GC test (Goldman 1993) has also been assessed. The GC metrics have been used to compare GTR+G_4_-based EMs to GTR+G_4_, GTR+I+G_4_, GTR, HKY+I+G_4_, HKY+G_4_, HKY, K80+I+G_4_, K80+G_4_, and K80-based SMs. Based on lack of resolution (table 4) observed in these experiments, comparisons involving other models have not been conducted. In order to obtain model-based likelihood values, RAxML searches were run for replicates generated under the above models as described above, specifying the general definition of the substitution model scheme (e.g., GTR+G_4_) and the correct full topological constraint which was used to create the corresponding replicate. The corresponding RAxML options led to adjustment of branch lengths and model numerical parameters during the program run.

To obtain the GC test statistics (*δ* = Ln(multinomial)−Ln(model)), the model-based likelihood values obtained have been compared with the corresponding unconstrained likelihood values which were calculated for the replicates. The GC test statistics for each EM-based replicate was compared with the SM-based distributions of the corresponding values to compute the absolute values of the *Z*-scores (numbers of standard deviations between the *δ* value for a EM replicate and the mean *δ* value for each SM-based replicate distribution).

The mean *Z*-scores over 500 individual tests for each EM-SM pair of evolutionary models were compared and the MS values for the resolution of the preferred models were calculated as described above. The MS values and the mean *Z*-scores were used to assess if the specification of the correct tree topology led to the best estimates of model fit within the 15-component SM models sets, each characterized by the same substitution model. The results have been summarized in table 4.

#### Comparison of Individual Test Values

Computation of the final test statistics (eq. 16), computation of all the MS values and the mean test values helping to summarize the discriminating ability of the tests compared here have been conducted with the help of test_stage2.pl script (available at: https://github.com/vadimgoremykin/absolute-model-data-fit).

### An Empirical Example

Empirical tests have been conducted based on a subset of a concatenated alignment of mammal nuclear genes presented by Song et al. (2012). Although large (1,297,456 aligned columns), the alignment presented by the authors (available from the DataDryad database) has only 10,042 columns which do not contain missing or ambiguous characters. In an attempt to increase the length of the alignment subset which can be used by the presented test, I have removed five species from the original file and produced a longer alignment partition which contains only DNA alphabet-specific characters. The partition, henceforth referred to as “empirical data set,” is available as Supplementary Material online. This data set has 32 OTUs and 29,900 aligned positions. The comparison of the aforementioned replicates to a shorter mitochondrial data set was not conducted because it contained many indels and missing genes/gene regions. The test presented here requires presence of only alphabet-specific characters in each alignment.

The methodological purpose of the experiments presented in this section was to 1) illustrate sampling of conflicting trees necessary to conduct the test, 2) to check for uniformity of the test results under different P-factor values in comparison to an implicit biological model, and 3) to check if the test rejects an obviously wrong evolutionary scenario. An additional goal was to identify the best-fitting model.

Five cladograms (provided in supplementary fig. S2, Supplementary Material online) used to generate model trees were selected based on the results of unconstrained phylogeny reconstruction experiments performed on the empirical data set. Tree reconstruction experiments assuming a CAT+GTR model with rates among sites modeled via a Dirichlet process (henceforth referred to as “CAT+GTR+D” model) were performed with the help of Phylobayes v. 3.3 (Lartillot et al. 2009). Maximum likelihood-based tree phylogeny reconstruction experiments were performed using IQ-TREE (v. 1.6, Nguyen et al. 2015). Neighbor-joining trees were built with the help of Seaview alignment editor (Gouy et al. 2010). The cladogram 1 corresponds to the tree obtained under the GTR model with rates across sites heterogeneity modeled via FreeRate model (Soubrier et al. 2012), which could be specified as GTR+R4 in IQ-TREE command line. The above-mentioned FreeRate model assuming four rate classes was selected as the best-fitting under the Bayesian Information Criterion (BIC) by the ModelFinder (Kalyaanamoorthy et al. 2017) pipeline run under specification of empirical data set as input alignment file. The cladogram 2 corresponds to the tree obtained under the GTR+G model. The cladograms 3 and 4 represent alternative consensus topologies recovered from two chains run under CAT+GTR+D model by discarding the first 500 cycles as “burn-in”—which was found to be sufficient for both chains—and building consensus trees based on next 1,500 cycles. The cladograms 1–4 differ only in placement of the root of placental mammals and in placement of tree shrew. The tree shrew appears as sister to primates on cladograms 1 and 2 and as sister to the rest of Euarchontoglires, a group which unites tree shrews, primates, lagomorphs, and rodents, on cladograms 3 and 4. The Atlantogenata (a group which includes Xenarthra and Afrotheria) appears as sister to the rest of placental mammals in the cladograms 1 and 3, and Xenartra assumes this position in cladograms 2 and 4.

The cladogram 5 represents the Neighbor-Joining tree topology which was recovered from Jukes and Cantor, K80 and LogDet distances using the Seaview alignment editor. This tree topology was included in analyses to provide an example of an obviously incorrect evolutionary hypothesis assuming rodents as the sister group to the rest of placentals (Churakov et al. 2010; Goremykin et al. 2010). The expectation was that the test would indicate a poor fit for this case.

Model parameters for the replicate generation under the optimal ML substitution model (GTR+R4) were determined in IQ-TREE searches run based on the empirical data set under iterative specification of each of the five above-mentioned topological constraints. Five hundred replicates were generated for each evolutionary model. The model-based replicate distributions obtained were compared with the empirical data set to assess absolute model fit employing P-factor values (eq. 8) 1,000, 10,000, and 100,000.

## Results

### Comparison of Explicitly Postulated Models

#### Identification of the Correct Substitution Models

The ability of the novel test to correctly identify the optimal substitution model among a set of alternatives was assessed, since arguably this is the most common goal of model fit-based investigations in the evolutionary studies. I checked if, under the correct tree topology specification for all evolutionary models compared, the SM showing the best fit to EM-based replicates would be the one that shares the same substitution model scheme with the EM. A less obvious hypothesis was also tested. This was that, within a set of SMs assuming different substitution model components and the same wrong specification of a model tree topology, the SM assuming the same substitution model scheme as the EM would show the best fit. The series of experiments performed to check all above hypotheses involved comparisons of a set of 10 SMs having 10 different substitution model components, all iteratively assuming model tree topologies 1 to 15 to each of 30 EMs.

In all these experiments, which have been conducted by comparing the mean values over 500 estimations of model-data fit of each SM (represented by a distribution of 500 replicates) to each of 500 EM-based replicates, the test presented here identified the SMs sharing the same substitution model with EMs as the best fitting model without a single exception.

These preliminary observations indicated that the novel test employed in this study was able to register an improvement in model-data fit due to specification of the correct substitution model component of the full evolutionary models under the selected experimental setup. Having checked this aspect of performance of the test, I proceeded to determine whether the test had sufficient discriminatory power to show improvement in model-data fit for the correct model tree topology.

#### Identification of the Correct Full Evolutionary Models

Simulated data sets for 150 full evolutionary models, including 10 distinct substitution model schemes, were iteratively compared with each of the 30 EMs. In all cases, the lowest mean test scores—estimated over 500 comparisons of each SM to each of 500 replicates simulated under each EM—identified the correct SMs without error. Had the test results been random, the probability of obtaining such results (30 correct identifications, each time out of 150 models) would have been 1/150^30^ = 5.2×10^−66^.

MS values were calculated that indicate the percentage of times when a correct SM, sharing the same tree and substitution model with EM, showed better fit to each of 500 replicates in comparison to any other distinct SM. The lowest MS values for each EM obtained in these experiments (table 1) ranged from 99% to 85% with the mean value 92%. Thus, the proposed test metric was also able to register the positive influence of the correct tree topology and of the correct substitution model scheme onto model-data fit estimates.

#### Identification of the Correct Tree Topology

Each of the 30 EMs was iteratively compared with 10 SM sets (300 comparisons in total). Each SM set included 15 full evolutionary models sharing a distinct (correct or misspecified) substitution model scheme. In all 300 experiments, the lowest mean test scores—estimated over 500 comparisons of each SM to each of 500 replicates simulated under each EM—identified the SMs assuming correct tree topologies.

The MS values indicating the percentage of times when each SM having correct or wrong substitution model component which assumed the correct tree topology (a “preferred SM”) showed better fit to each of 500 replicates simulated under a distinct EM in comparison to any other SM assuming the same substitution model scheme as the preferred SM have been also computed in these experiments. The results of these comparisons are presented in table 2. The MS values in the table 2 show the worst measure of separation between each preferred SM and any other SM which has the same substitution model component and a wrong tree component registered in these experiments.

The above results indicate that the proposed test run employing default P-factor value of 10,000 was able to detect an improvement in the model-data fit due to a correct tree topology specification. This was indicated by the MS values for each preferred model (shown in table 2) regardless of the substitution model misspecification for all the models used in the experiments. The chance of correct model tree component identification for the models showing the highest level of misfit was not much different compared with the models exhibiting no substitution model misspecification (table 2). The chance of identifying the correct model tree component estimated at P-factor values set to 10,000 and 100,000 for a subset of models were identical and very similar to the results obtained employing a P-factor value = 1,000 (table 3).

Comparisons were also made using GC test metric. In these experiments, the MS values for the preferred models obtained were generally low, and in many cases produced the lowest possible value (zero) (table 4). This indicates that the discriminatory power of the GC test was not sufficient to reliably reveal the improvement in the model-data fit due to the choice of the correct model tree topology, even in the case in which the SM substitution model components were correctly specified.

### Testing Evolutionary Relationships among Mammals

**Fig. 4.**
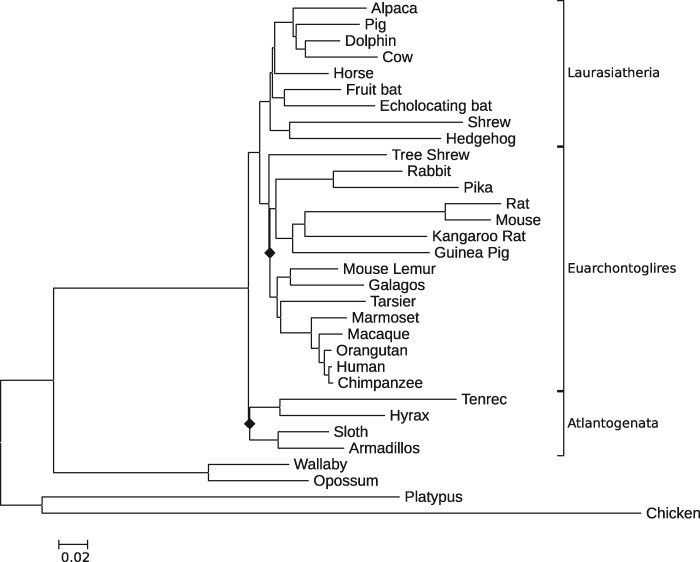
—The phylogenetic relationships among the taxa in empirical data set supported by the test. Shown is a tree recovered with the help of Phylobayes under CAT+GTR+D model. The PP support values for the branches marked with diamonds were low (<0.6). Other PP support values were equal to 1. The branch lengths are represented by a scale bar (bottom left).

The test identified as best-fitting to the empirical data set the evolutionary model assuming the tree topology presented in supplementary figure S2 (Supplementary Material online) as cladogram 3. The topology corresponds to a tree (shown in fig. 4) recovered under a CAT+GTR+D model using Phylobayes v. 3.3 program (Lartillot et al. 2009).

The tree topology supports the clade subtending Atlantogenata as sister to the rest of the placental mammals and the clade subtending tree shrew as sister to the rest of the Euarchontoglires. This result was obtained with P-factor values (eq. 8) ranging in size by two orders of magnitude: 1,000, 10,000, and 100,000. The ranking of evolutionary models under the test metric using the above P-factor values was identical (in descending order of fit estimated for the evolutionary models assuming full topological constraints shown in supplementary fig. S2: 3, 4, 1, 2, 5, Supplementary Material online). The model-data fit estimates (table 5) for each evolutionary model obtained employing the above P-factor values were very similar.

It should be noted that the worst assessment of model-data fit was registered for the evolutionary model which assumed model tree topology 5 supporting rodents as sisters to the rest of the placental mammals. The placement of rodents at the base of the eutherian subtree is considered a classical example of LBA (Churakov et al. 2010; Goremykin et al. 2010).

## Discussion

Using data to test a model which is assumed to describe its generation is an important principle in statistical analysis. Absolute model-data fit assessment showing how well simulated data fit the observed can be used to quantify model strengths and weaknesses and can be used as a guide for model improvement. However, absolute tests of data model fit are rarely used in the field of phylogenetics. This is arguably so because the task of identification of the correct evolutionary tree, which is fundamental for the discipline, is reportedly difficult to accomplish using previous model-data fit indicators. Concerns about their discriminatory power were raised more than a decade ago (Foster 2004; Waddell et al. 2009; Ripplinger and Sullivan 2010) yet no method of model-data fit assessment, able to reliably discriminate phylogenetic hypotheses assuming different trees, has been forthcoming.

The discriminatory power of model-data fit indicators strongly depends on which aspects of the data are used to compare models. Here, I tested how well test statistics derived from distributions of characters in observed and simulated data were able to detect phylogenetic signal due to treelike divergence of sequences.

In the experiments performed here, the estimator based on the multinomial likelihood failed to reliably identify the correct model trees under the easiest conditions (assuming availability of the correct empirical model and no substitution model scheme misspecification). The evolutionary models selected by the GC test as the most adequate included wrong model trees as their components in the vast majority of analyses (table 4). These observations indicate that multinomial likelihood-based statistic derived from the frequencies of site patterns is poorly suited to detect tree-based phylogenetic signal in the multiple sequence alignments. Since the phylogenetic information contained in the patterns themselves is irrelevant for multinomial likelihood inference, the GC test statistic does not capture the data properties which are relevant for tree building.

The main advantage of the novel test of model-data fit proposed here over the GC test is its accuracy. However, for its successful application certain conditions should be fulfilled. The test presented here was designed to enable discrimination of the distinct, fully specified evolutionary models. Each such model should assume a fixed parameter combination and a fully resolved tree with fixed branch lengths. In order to allow for meaningful model comparison among the evolutionary hypotheses, all model parameters should assume values registered at the ML optimum under each evolutionary model specification. Also, the test accuracy observed in comparison of explicitly postulated models was partially due to the fact that the multiple sequence alignments compared (represented by replicates) had no alignment errors. When the observed alignment contains character states not predicted by DNA substitution models, these must be removed before the method can be used for model evaluation. In this case, a necessary data preparation step might include, in any combination, removal of corresponding sequences and/or removal of sites. The preferred trimming scheme should be selected by an analyst to fit the purpose of the study prior to analysis.

Under such conditions the novel estimator has shown an ability to identify the correct evolutionary models. The probability that this result can be explained by a tolerance of the experimental setup to a random error was small (5.2×10^−66^).

Under the conditions studied, this test statistic identified the correct model tree in cases where the substitution model was correctly specified and also misspecified. The ability to identify the true tree topology within broad margins of model misspecification observed here is encouraging that correct identification of the best-fitting evolutionary model is possible when the correct model is unknown (as might be the case with biological data). Some degree of model misfit is unavoidable when substitution process in biological data is modeled.

Nevertheless the robustness of the test’s results in the presence of model misspecification does not remove the requirement for realistic SM substitution models in the analyses of the biological data. When EM is unknown or implicit the tree representing the preferred evolutionary hypothesis should be selected as a component of the best-fitting SM.

The discriminatory power of the test reported here encourages the application of the novel test for hypothesis testing in phylogenetics. Given the rarity with which assessment of absolute model-data fit are currently employed in the field, its introduction into the mainstream phylogenetic practice would help to diminish the gap between a large number of phylogenetic relationships claimed to be correctly resolved and the ancillary indicators (bootstrap support, posterior probability support, and recovery of congruent trees with different methods) often used to corroborate such claims. I hope that the observations made in this study can help to develop more realistic substitution models and to help with efforts to test and more reliably reconstruct the Tree of Life.

Concluding, I would like to mention that the results obtained here in analyses involving empirical data support Atlantogenata as the basal-most clade among placental mammals and sister group relationship between tree shrews (*Tupaia*) and the rest of Euarchontoglires. The former phylogenetic relationship has often been recovered in recent studies (e.g., Song et al. 2012; Morgan et al. 2013; Tarver et al. 2016). The latter relationship has been recently recovered by Tarver et al. (2016) based on a data set comprising microRNA genes and in coalescent analyses presented by Mason et al. (2016).

## Supplementary Material

Supplementary data are available at *Genome Biology and Evolution* online.

## Supplementary Material

evz167_Supplementary_Data
