# Supplementary material for: A Novel Test for Absolute Fit of Evolutionary Models Provides a Means to Correctly Identify the Substitution Model and the Model Tree
Source: Genome Biol Evol. 2019 Aug 1;11(8):2403–19. doi: 10.1093/gbe/evz167 (PMC6736042; doi:10.1093/gbe/evz167)
Supplement: evz167_Supplementary_Data [file evz167_supplementary_data.zip › SUPPLEMENTARY_MATERIAL.tgz!/SUPPLEMENTARY_MATERIAL/Supplementary_figure_S2.pdf]

Supplementary figure S2. Cladograms used to generate model trees in analyses of empirical data. The branch subtending Xenarthra is highlighted in fuchsia. The branch subtending Afrotheria is highlighted in orange. The branches subtending Lagomorpha and Rodentia are highlighted, respectively, in light and dark blue. The branch subtending primates is highlighted in red, and the one subtending tree shrew is highlighted in green.

Cladogram 1

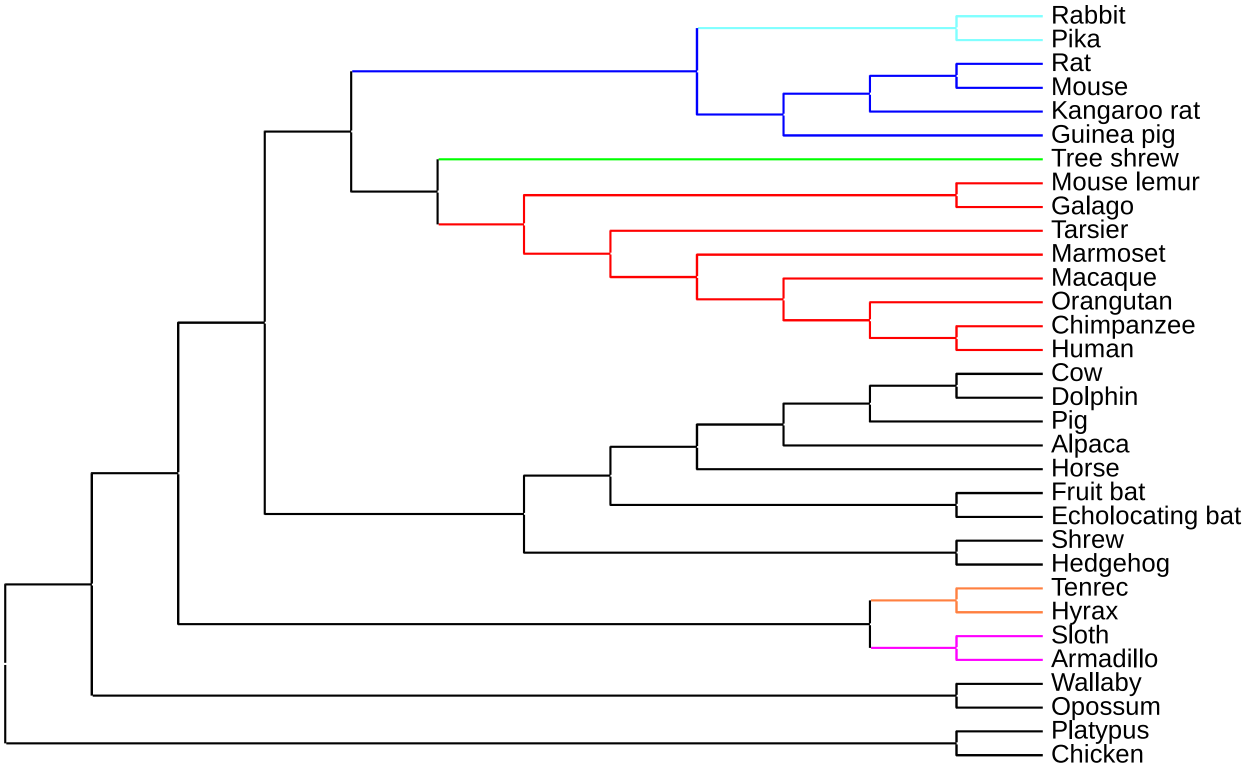

Cladogram 2

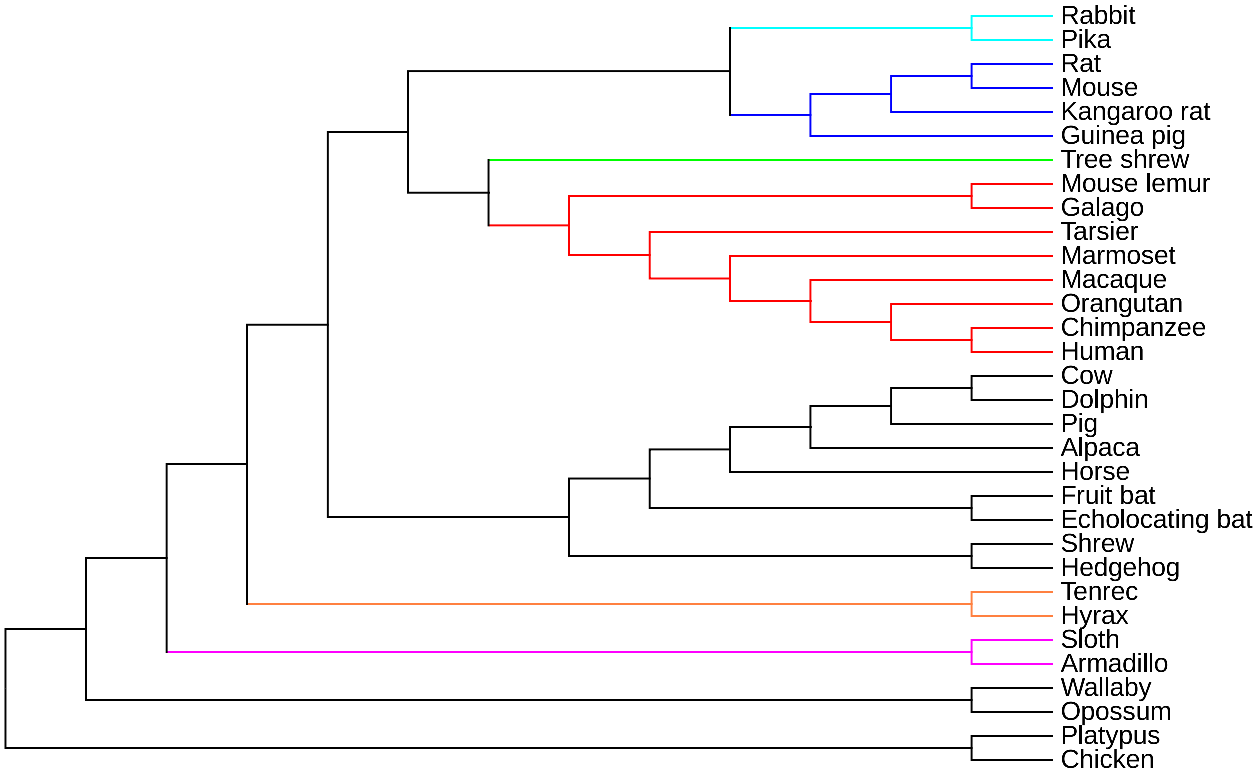

Cladogram 3

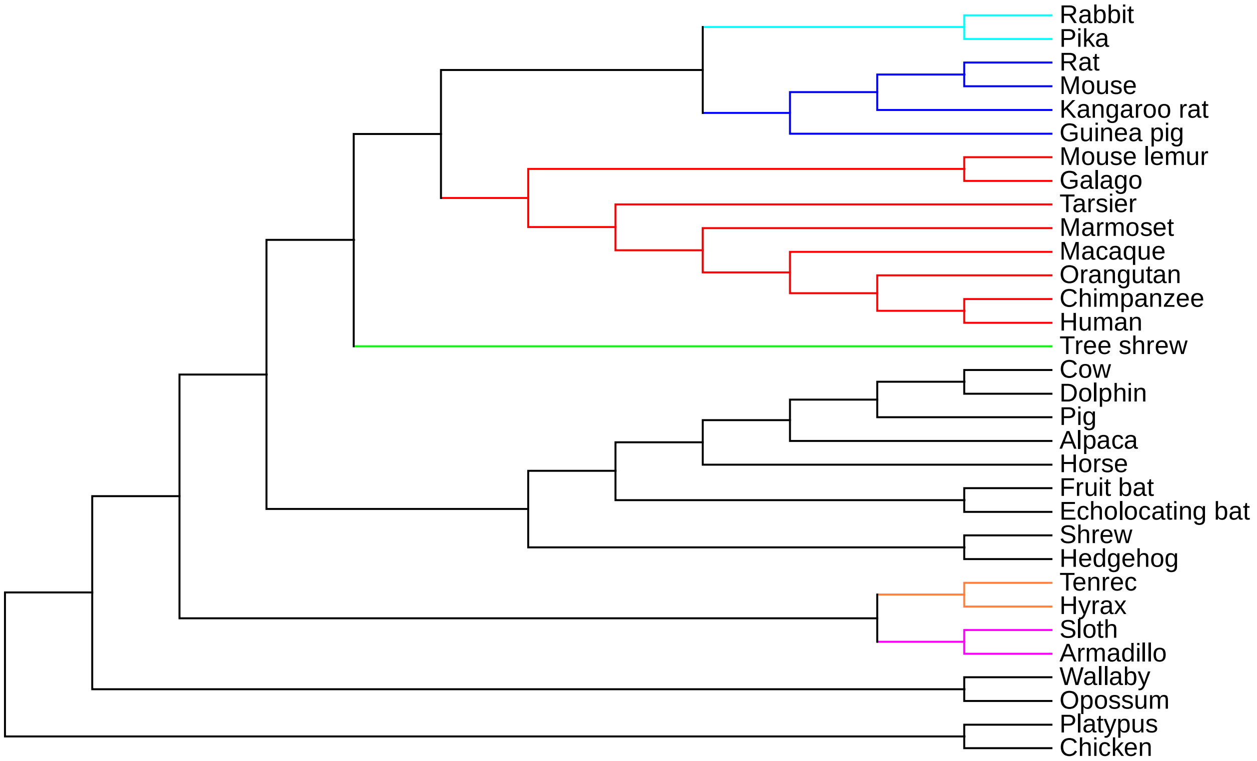

Cladogram 4

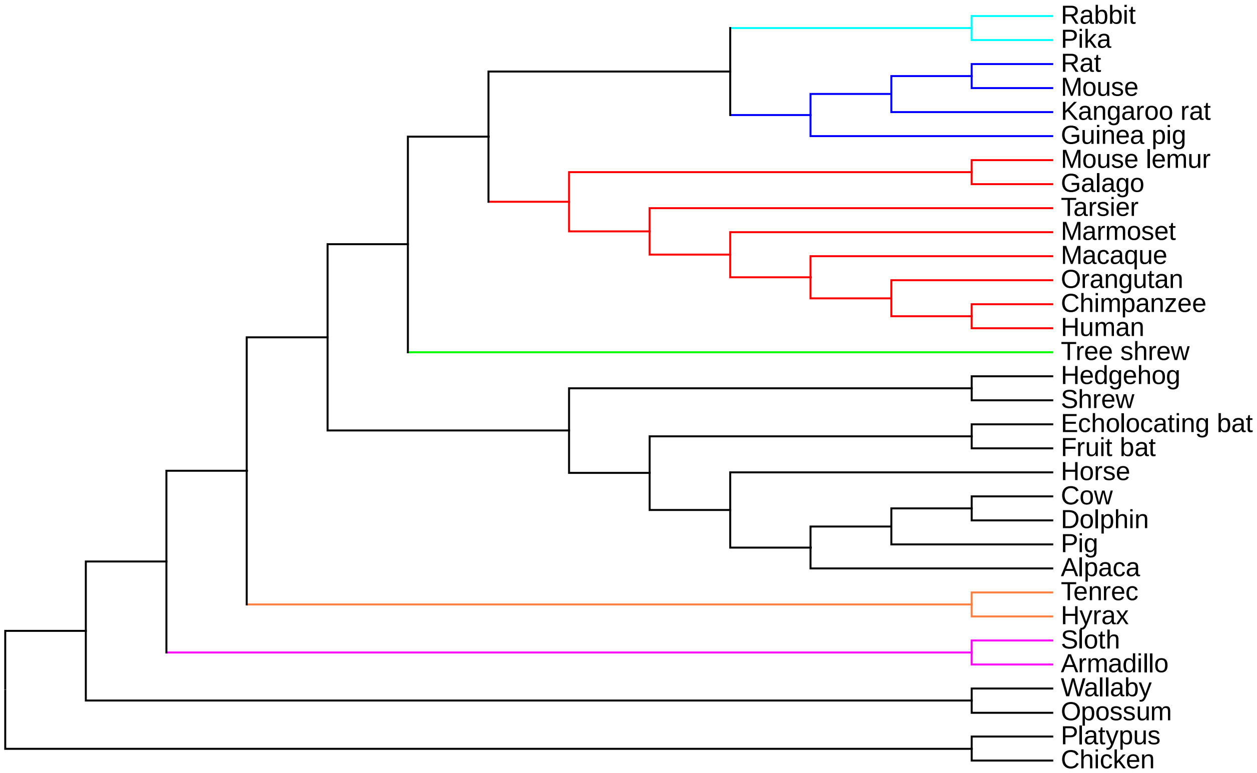

Cladogram 5

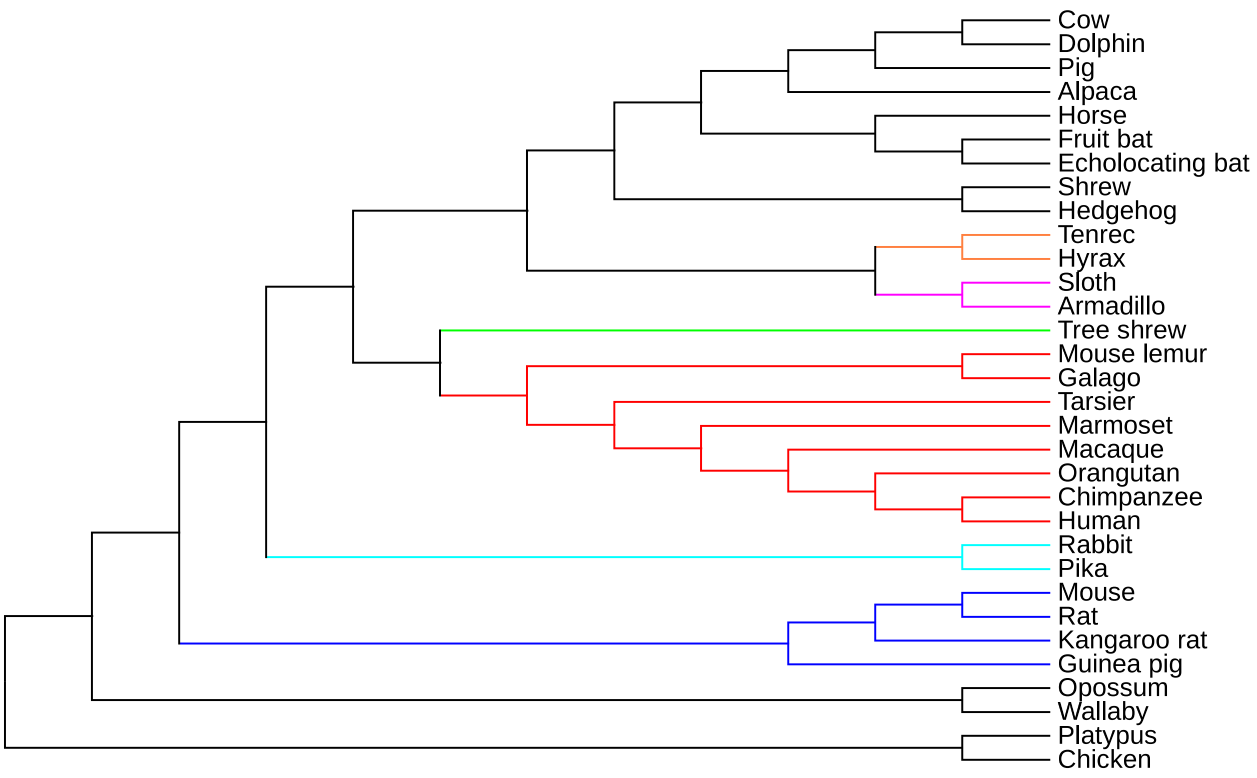
Cow
Dolphin
Pig
Alpaca
Horse
Fruit bat
Echolocating bat
Shrew
Hedgehog
Tenrec
Hyrax
Sloth
Armadillo
Tree shrew
Mouse lemur
Galago
Tarsier
Marmoset
Macaque
Orangutan
Chimpanzee
Human
Rabbit
Pika
Mouse
Rat
Kangaroo rat
Guinea pig
Opossum
Wallaby
Platypus
Chicken
