# Supplementary material for: A Novel Test for Absolute Fit of Evolutionary Models Provides a Means to Correctly Identify the Substitution Model and the Model Tree
Source: Genome Biol Evol. 2019 Aug 1;11(8):2403–19. doi: 10.1093/gbe/evz167 (PMC6736042; doi:10.1093/gbe/evz167)
Supplement: evz167_Supplementary_Data [file evz167_supplementary_data.zip › SUPPLEMENTARY_MATERIAL.tgz!/SUPPLEMENTARY_MATERIAL/Supplementary_figure_S1.pdf]

Supplementary figure S1. Model tree topologies used in the comparisons of explicit models. The branches rearranged in the trees are color-highlighted. The corresponding leaf labels are highlighted in bold for the land plants and in bold italics for streptophyte algae. The branch lengths shown correspond to the tree components of GTR+G-based empirical models.

Tree 1

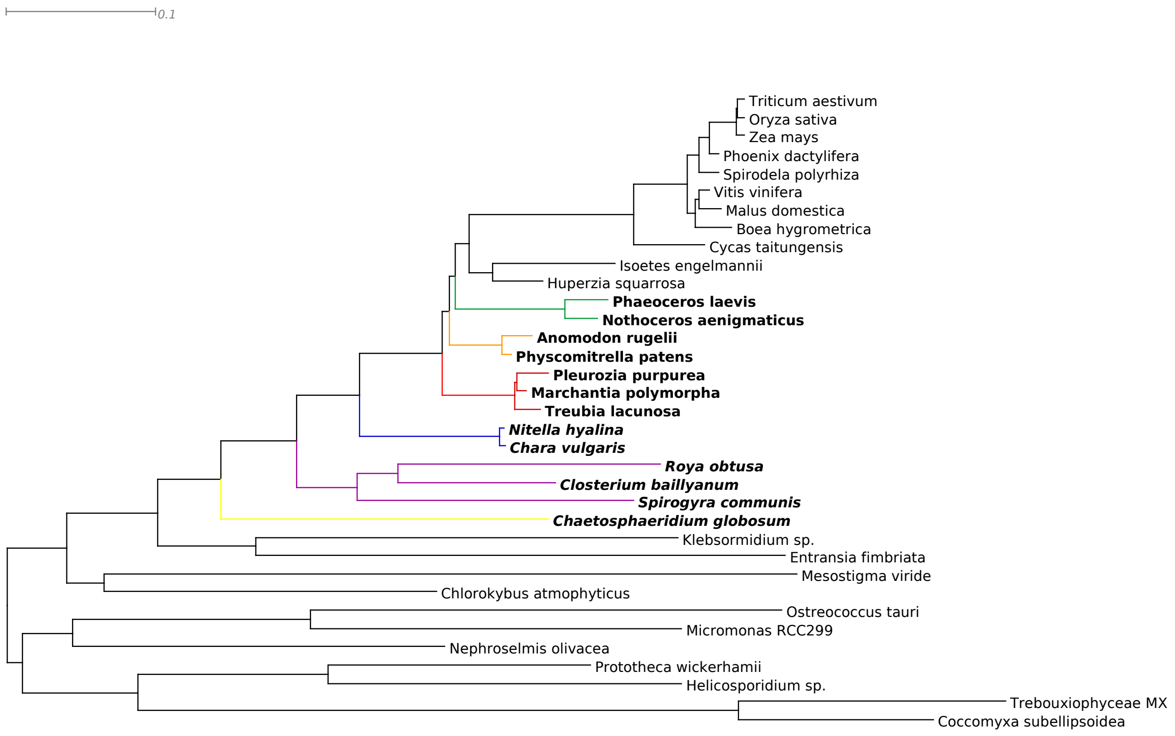

Tree 2

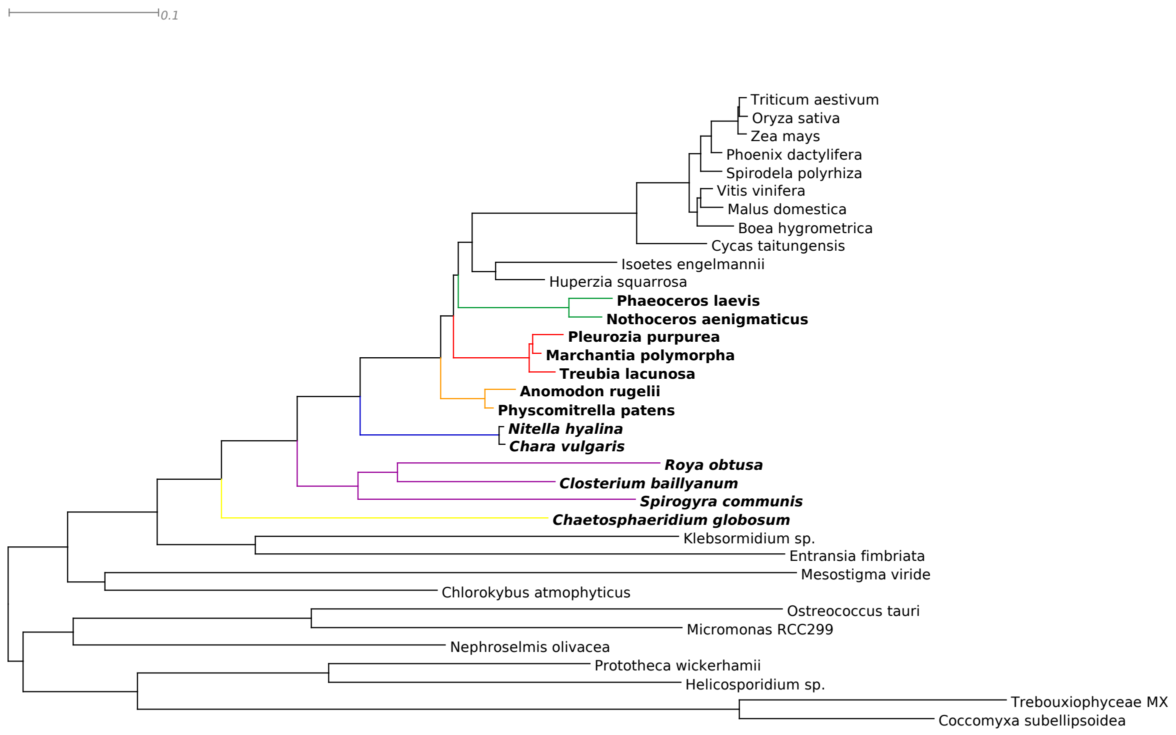

Tree 3

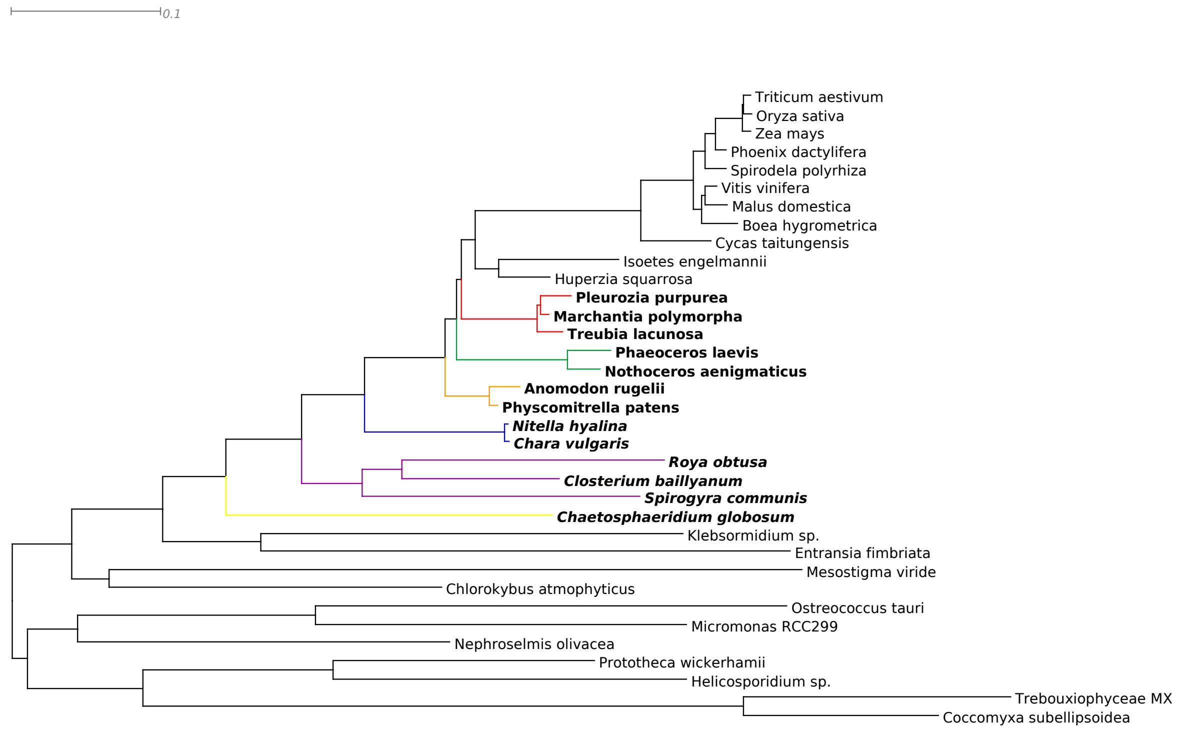
0.1
Triticum aestivum
Oryza sativa
Zea mays
Phoenix dactylifera
Spirodela polyrhiza
Vitis vinifera
Malus domestica
Boea hygrometrica
Cycas taitungensis
Isoetes engelmannii
Huperzia squarrosa
Pleurozia purpurea
Marchantia polymorpha
Treubia lacunosa
Phaeoceros laevis
Nothoceros aenigmaticus
Anomodon rugelii
Physcomitrella patens
Nitella hyalina
Chara vulgaris
Roya obtusa
Closterium baillyanum
Spirogyra communis
Chaetosphaeridium globosum
Klebsormidium sp.
Entransia fimbriata
Mesostigma viride
Chlorokybus atmophyticus
Ostreococcus tauri
Micromonas RCC299
Nephroselmis olivacea
Prototheca wickerhamii
Helicosporidium sp.
Trebouxiophyceae MX
Coccomyxa subellipsoidea

Tree 4

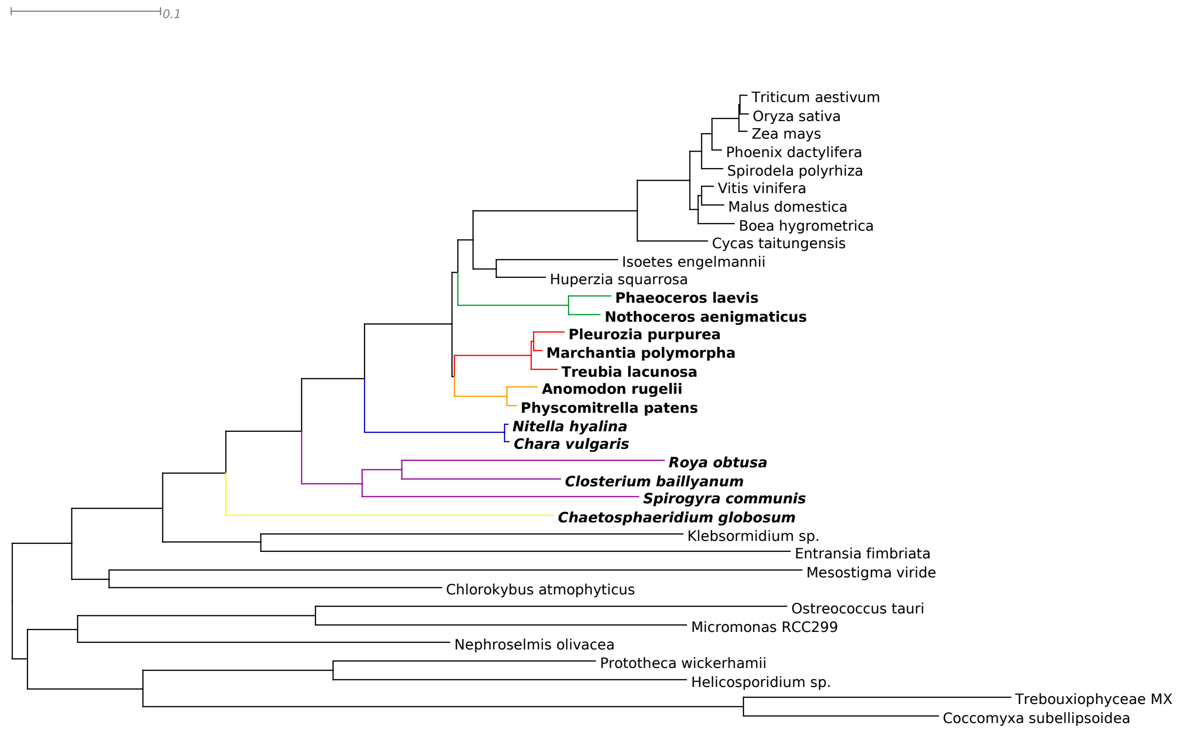
0.1
Triticum aestivum
Oryza sativa
Zea mays
Phoenix dactylifera
Spirodela polyrhiza
Vitis vinifera
Malus domestica
Boea hygrometrica
Cycas taitungensis
Isoetes engelmannii
Huperzia squarrosa
Phaeoceros laevis
Nothoceros aenigmaticus
Pleurozia purpurea
Marchantia polymorpha
Treubia lacunosa
Anomodon rugelii
Physcomitrella patens
Nitella hyalina
Chara vulgaris
Roya obtusa
Closterium baillyanum
Spirogyra communis
Chaetosphaeridium globosum
Klebsormidium sp.
Entransia fimbriata
Mesostigma viride
Chlorokybus atmophyticus
Ostreococcus tauri
Micromonas RCC299
Nephroselmis olivacea
Prototheca wickerhamii
Helicosporidium sp.
Trebouxiophyceae MX
Coccomyxa subellipsoidea

Tree 5

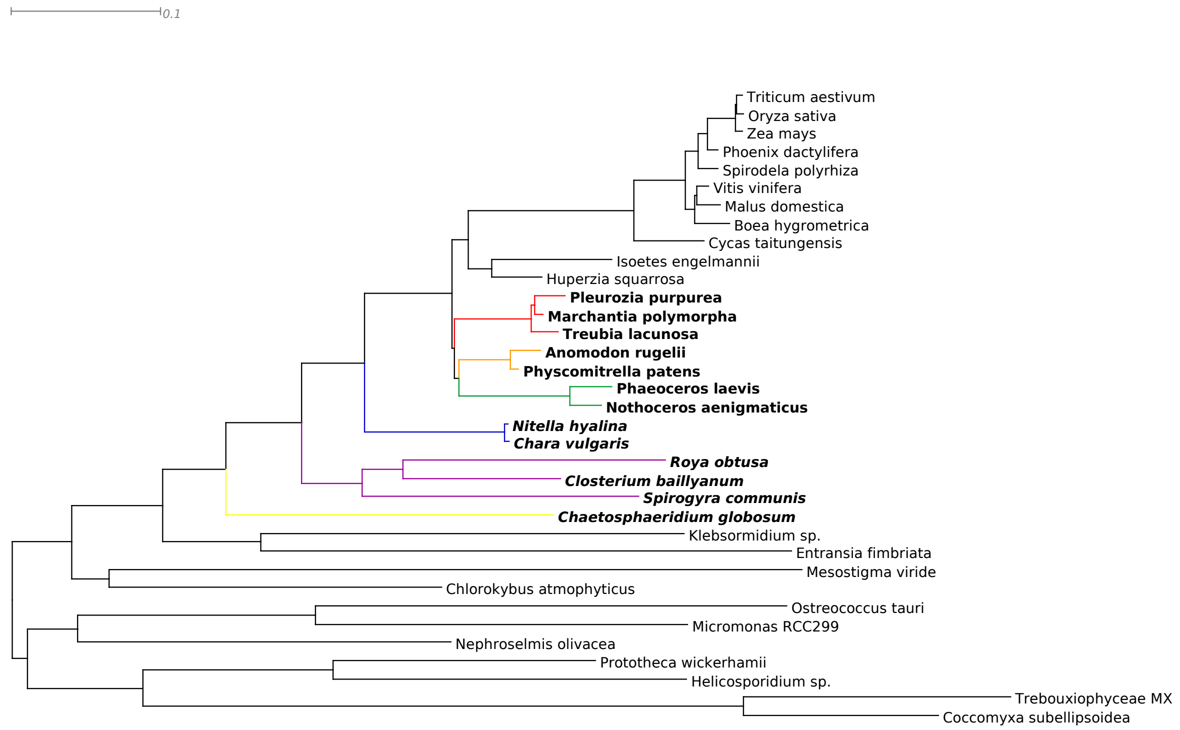
0.1
Triticum aestivum
Oryza sativa
Zea mays
Phoenix dactylifera
Spirodela polyrhiza
Vitis vinifera
Malus domestica
Boea hygrometrica
Cycas taitungensis
Isoetes engelmannii
Huperzia squarrosa
Pleurozia purpurea
Marchantia polymorpha
Treubia lacunosa
Anomodon rugelii
Physcomitrella patens
Phaeoceros laevis
Nothoceros aenigmaticus
Nitella hyalina
Chara vulgaris
Roya obtusa
Closterium baillyanum
Spirogyra communis
Chaetosphaeridium globosum
Klebsormidium sp.
Entransia fimbriata
Mesostigma viride
Chlorokybus atmophyticus
Ostreococcus tauri
Micromonas RCC299
Nephroselmis olivacea
Prototheca wickerhamii
Helicosporidium sp.
Trebouxiophyceae MX
Coccomyxa subellipsoidea

Tree 6

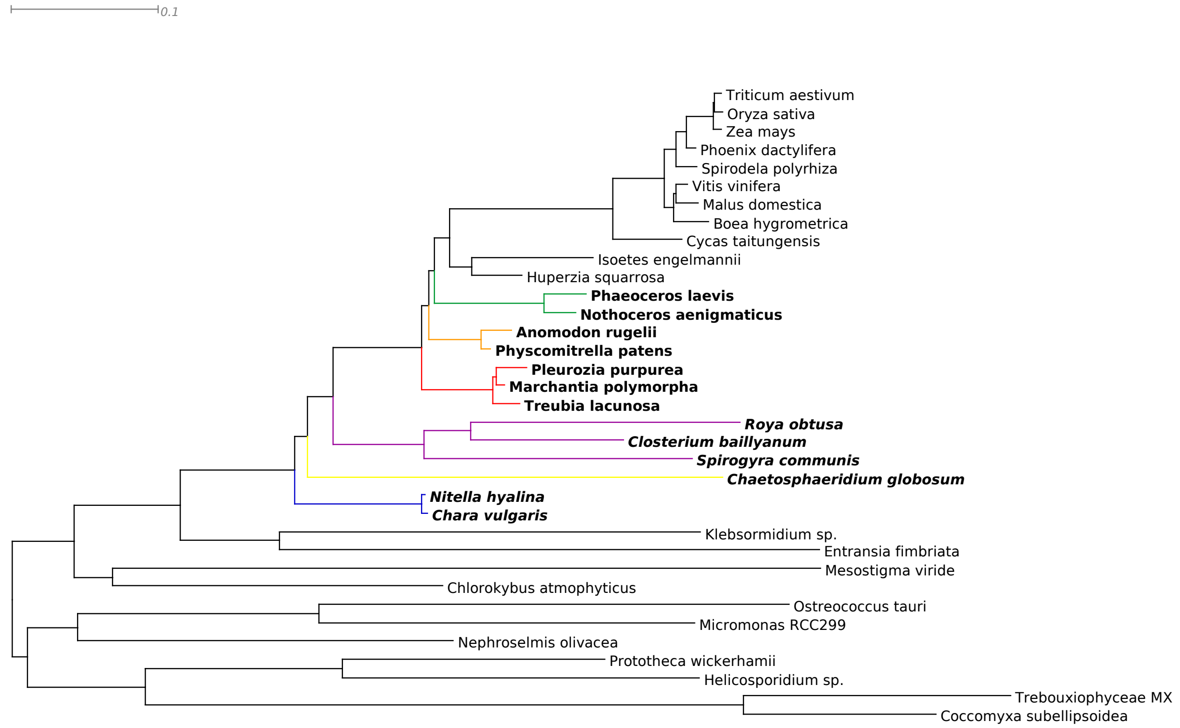
0.1
Triticum aestivum
Oryza sativa
Zea mays
Phoenix dactylifera
Spirodela polyrhiza
Vitis vinifera
Malus domestica
Boea hygrometrica
Cycas taitungensis
Isoetes engelmannii
Huperzia squarrosa
Phaeoceros laevis
Nothoceros aenigmaticus
Anomodon rugelii
Physcomitrella patens
Pleurozia purpurea
Marchantia polymorpha
Treubia lacunosa
Roya obtusa
Closterium baillyanum
Spirogyra communis
Chaetosphaeridium globosum
Nitella hyalina
Chara vulgaris
Klebsormidium sp.
Entransia fimbriata
Mesostigma viride
Chlorokybus atmophyticus
Ostreococcus tauri
Micromonas RCC299
Nephroselmis olivacea
Prototheca wickerhamii
Helicosporidium sp.
Trebouxiophyceae MX
Coccomyxa subellipsoidea

Tree 7

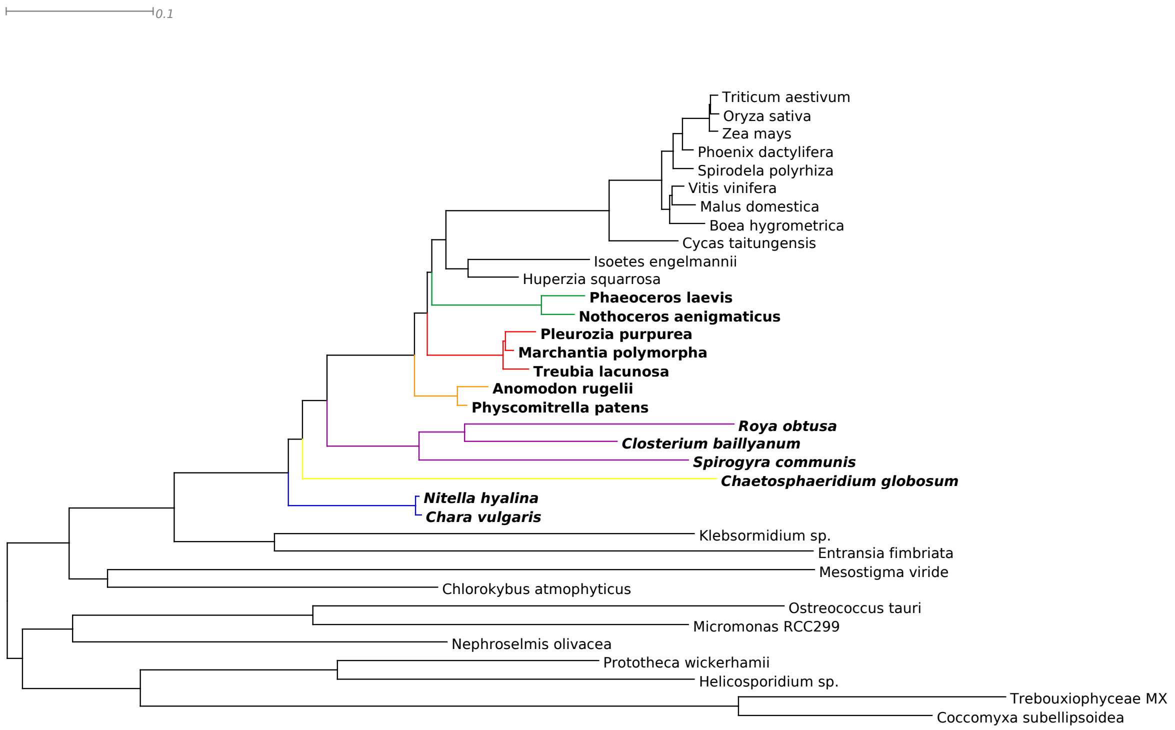

0.1
Triticum aestivum
Oryza sativa
Zea mays
Phoenix dactylifera
Spirodela polyrhiza
Vitis vinifera
Malus domestica
Boea hygrometrica
Cycas taitungensis
Isoetes engelmannii
Huperzia squarrosa
Phaeoceros laevis
Nothoceros aenigmaticus
Pleurozia purpurea
Marchantia polymorpha
Treubia lacunosa
Anomodon rugelii
Physcomitrella patens
Roya obtusa
Closterium baillyanum
Spirogyra communis
Chaetosphaeridium globosum
Nitella hyalina
Chara vulgaris
Klebsormidium sp.
Entransia fimbriata
Mesostigma viride
Chlorokybus atmophyticus
Ostreococcus tauri
Micromonas RCC299
Nephroselmis olivacea
Prototheca wickerhamii
Helicosporidium sp.
Trebouxiophyceae MX
Coccomyxa subellipsoidea

Tree 8

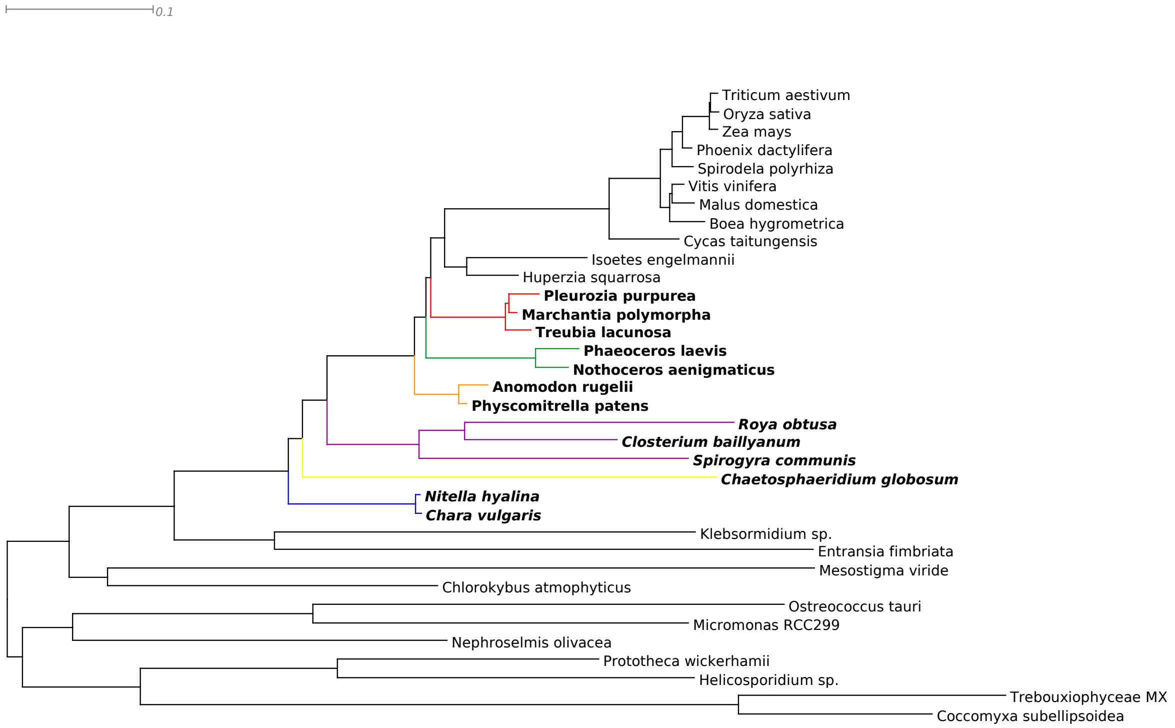

0.1
Triticum aestivum
Oryza sativa
Zea mays
Phoenix dactylifera
Spirodela polyrhiza
Vitis vinifera
Malus domestica
Boea hygrometrica
Cycas taitungensis
Isoetes engelmannii
Huperzia squarrosa
Pleurozia purpurea
Marchantia polymorpha
Treubia lacunosa
Phaeoceros laevis
Nothoceros aenigmaticus
Anomodon rugelii
Physcomitrella patens
Roya obtusa
Closterium baillyanum
Spirogyra communis
Chaetosphaeridium globosum
Nitella hyalina
Chara vulgaris
Klebsormidium sp.
Entransia fimbriata
Mesostigma viride
Chlorokybus atmophyticus
Ostreococcus tauri
Micromonas RCC299
Nephroselmis olivacea
Prototheca wickerhamii
Helicosporidium sp.
Trebouxiophyceae MX
Coccomyxa subellipsoidea

Tree 9

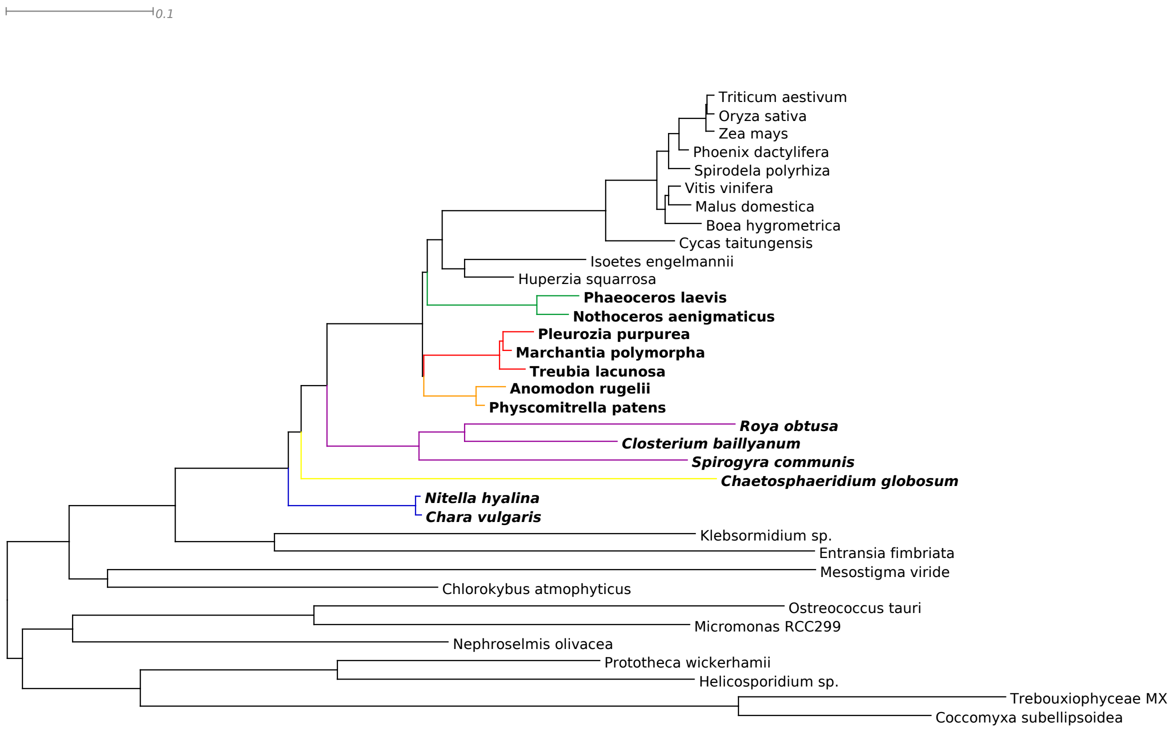

Tree 10

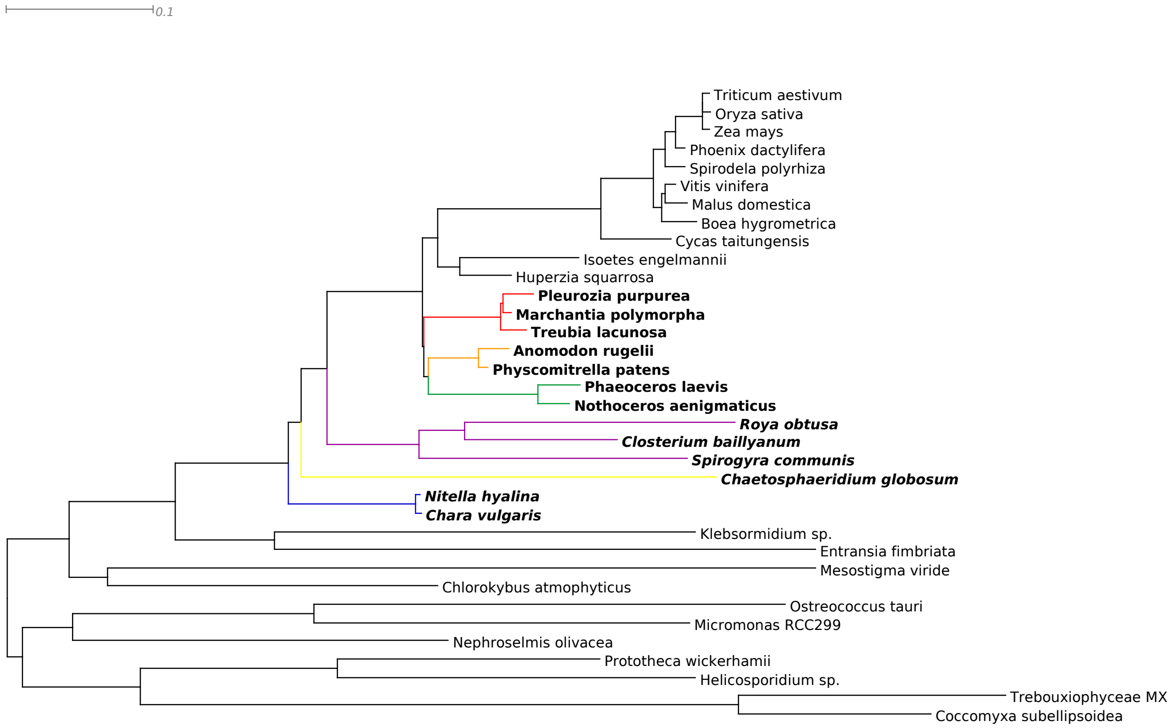

Tree 11

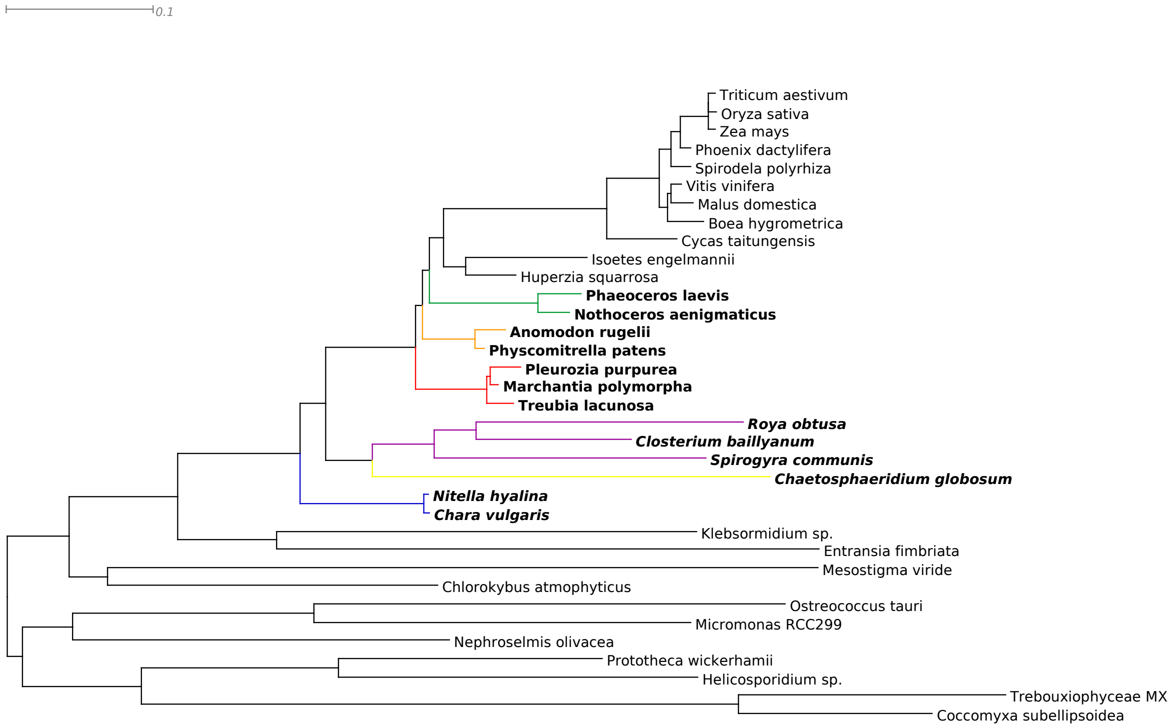

Tree 12

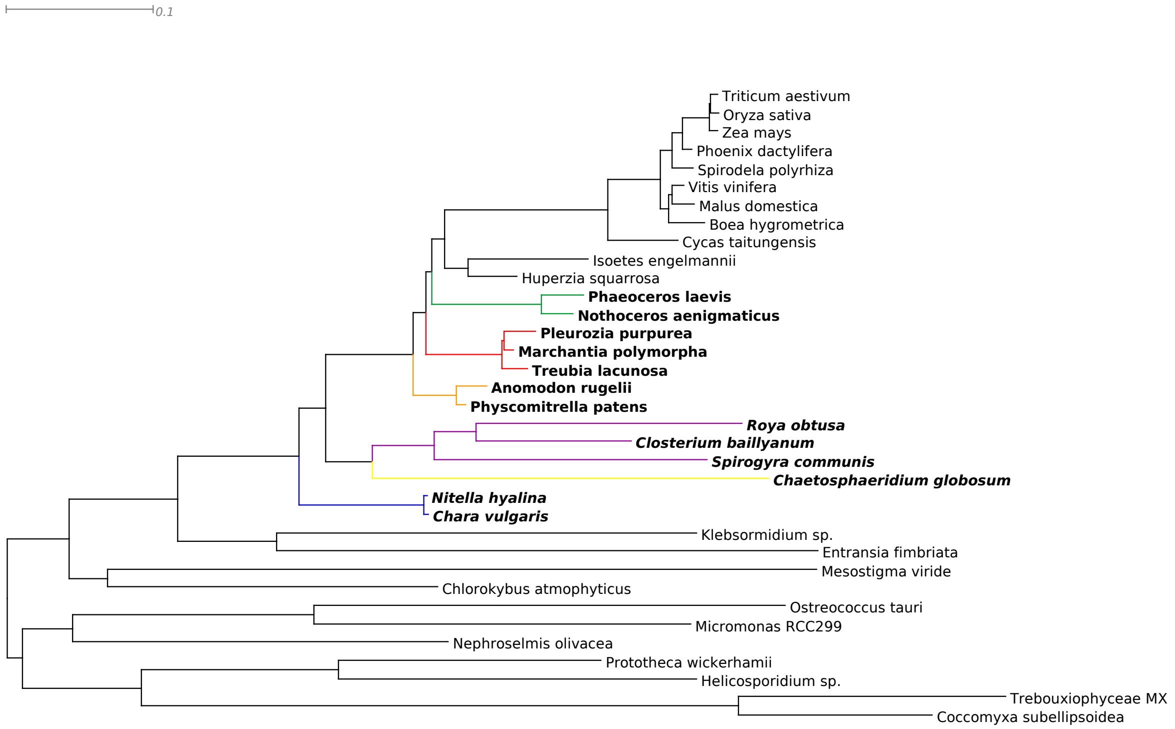

Tree 13

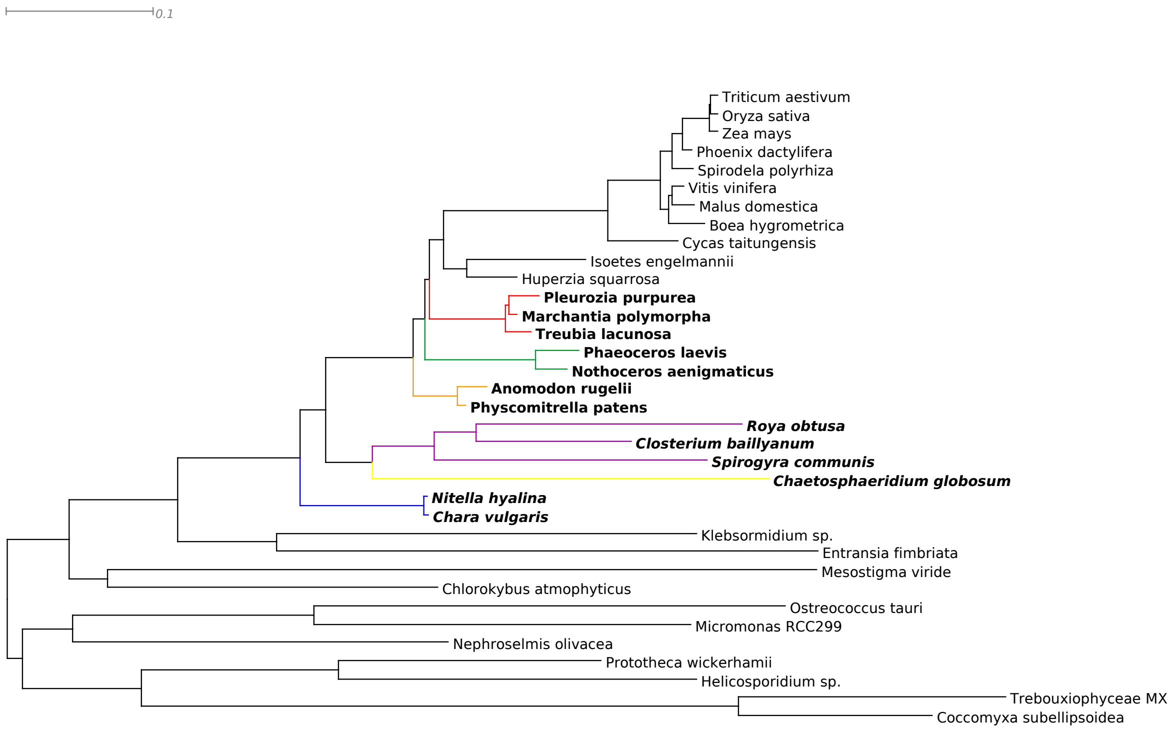

Tree 14

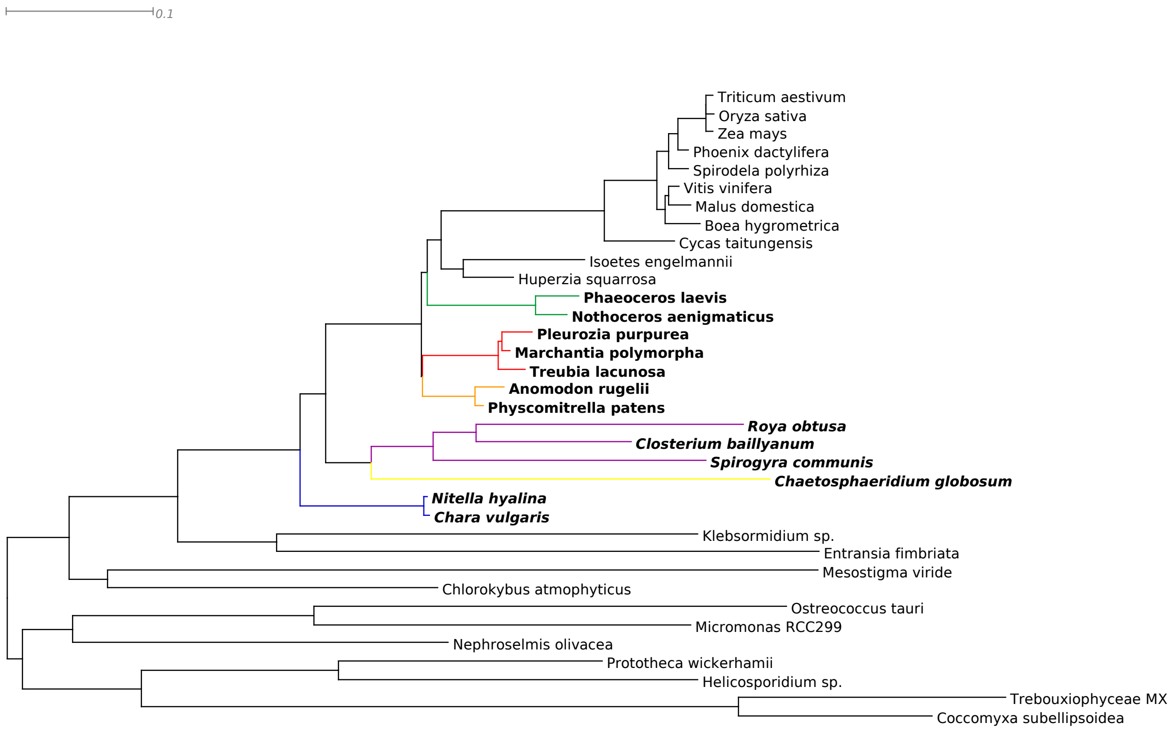

Tree 15

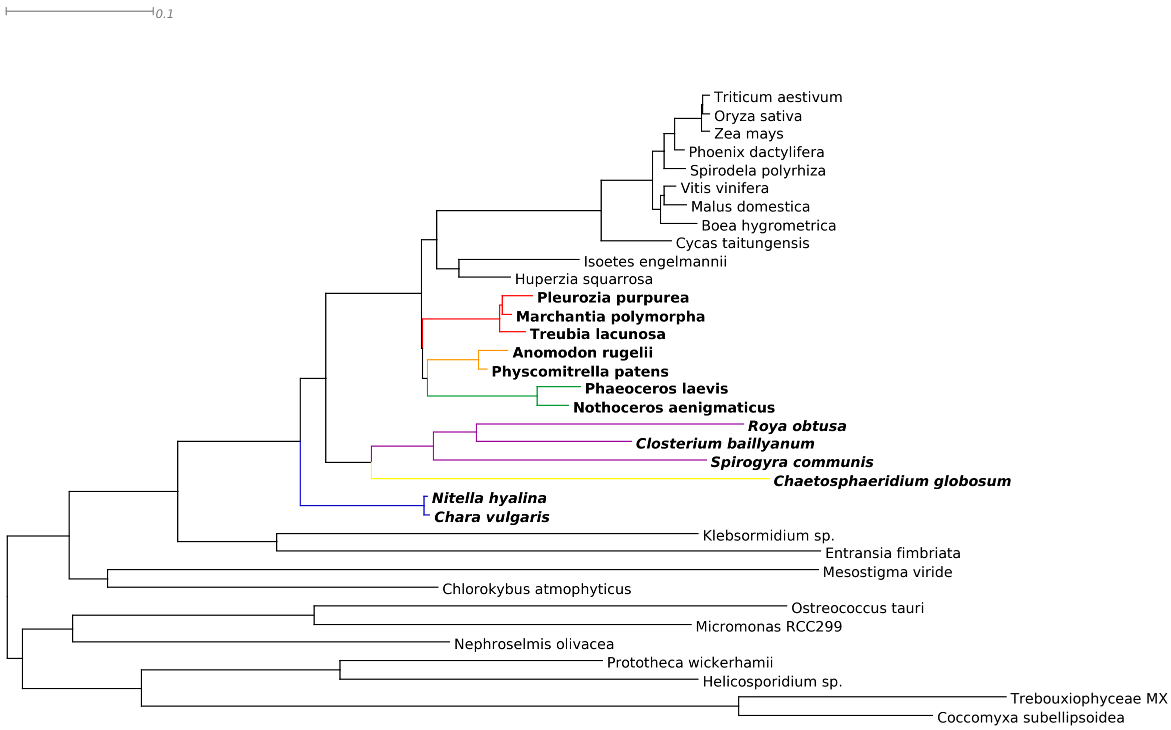

0.1
Triticum aestivum
Oryza sativa
Zea mays
Phoenix dactylifera
Spirodela polyrhiza
Vitis vinifera
Malus domestica
Boea hygrometrica
Cycas taitungensis
Isoetes engelmannii
Huperzia squarrosa
Pleurozia purpurea
Marchantia polymorpha
Treubia lacunosa
Anomodon rugelii
Physcomitrella patens
Phaeoceros laevis
Nothoceros aenigmaticus
Roya obtusa
Closterium baillyanum
Spirogyra communis
Chaetosphaeridium globosum
Nitella hyalina
Chara vulgaris
Klebsormidium sp.
Entransia fimbriata
Mesostigma viride
Chlorokybus atmophyticus
Ostreococcus tauri
Micromonas RCC299
Nephroselmis olivacea
Prototheca wickerhamii
Helicosporidium sp.
Trebouxiophyceae MX
Coccomyxa subellipsoidea
